# A High-Content Screen Reveals New Small-Molecule Enhancers of Ras/Mapk Signaling as Probes for Zebrafish Heart Development

**DOI:** 10.3390/molecules23071691

**Published:** 2018-07-11

**Authors:** Manush Saydmohammed, Laura L. Vollmer, Ezenwa O. Onuoha, Taber S. Maskrey, Gregory Gibson, Simon C. Watkins, Peter Wipf, Andreas Vogt, Michael Tsang

**Affiliations:** 1 Department of Developmental Biology, University of Pittsburgh, BST3, 3501 5th Avenue, Pittsburgh, PA 15213, USA; mas386@pitt.edu (M.S.); ezeobi.onu@gmail.com (E.O.O.); 2 The University of Pittsburgh Drug Discovery Institute, 200 Lothrop Street, Pittsburgh, PA 15260, USA; llv4@pitt.edu; 3 Department of Chemistry, 219 University Drive, University of Pittsburgh, Pittsburgh, PA 15260, USA; taber.maskrey@pitt.edu (T.S.M.); pwipf@pitt.edu (P.W.); 4 Department of Cell Biology, University of Pittsburgh, 3500 Terrace Street, Pittsburgh, PA 15213, USA; gregory.gibson@pitt.edu (G.G.); simon.watkins@pitt.edu (S.C.W.); 5 Department of Computational and Systems Biology, University of Pittsburgh, Pittsburgh, PA 15213, USA

**Keywords:** zebrafish, high-throughput screening, high-content analysis, Cognition Network Technology, Fgf signaling, heart development, probe discovery

## Abstract

Zebrafish is the preferred vertebrate model for high throughput chemical screens to discover modulators of complex biological pathways. We adapted a transgenic zebrafish line, *Tg(dusp6:EGFP)*, which reports on fibroblast growth factor (Fgf)/Ras/Mapk activity, into a quantitative, high-content chemical screen to identify novel Fgf hyperactivators as chemical probes for zebrafish heart development and regeneration. We screened 10,000 compounds from the TimTec ActiProbe library, and identified several structurally distinct classes of molecules that enhanced Fgf/Ras/Mapk signaling. We chose three agents—ST020101, ST011282, and ST006994—for confirmatory and functional studies based on potency, repeatability with repurchased material, favorable whole organism toxicity, and evidence of structure–activity relationships. Functional follow-up assays confirmed that all three compounds induced the expression of Fgf target genes during zebrafish embryonic development. Moreover, these compounds increased cardiac progenitor populations by effecting a fate change from endothelial to cardiac progenitors that translated into increased numbers of cardiomyocytes. Interestingly, ST006994 augmented Fgf/Ras/Mapk signaling without increasing Erk phosphorylation, suggesting a molecular mechanism of action downstream of Erk. We posit that the ST006994 pharmacophore could become a unique chemical probe to uncover novel mechanisms of Fgf signaling during heart development and regeneration downstream of the Mapk signaling node.

## 1. Introduction

One of the central goals of chemical genetics is to identify small molecule candidates that can modulate a biological pathway or a specific protein function. The use of living multicellular organisms is advantageous over cell-based assays or protein binding biochemical assays, as it allows the identification of unique developmental phenotypes and an analysis of biological processes in the proper context. Zebrafish at the embryonic or larval stages have become the favorite discovery model, because they are the only vertebrates that are compatible with high throughput screening [1,2,3,4,5,6]. Recent work has highlighted the applicability and successes of utilizing the zebrafish embryo as a vertebrate model for chemical screens. The phenotypes that zebrafish larvae can offer range from pancreas growth [7] to altered activity and behavior [8,9] through cancer cell metastasis [10] and organ regeneration [11], to name only a few. Our research is focused on the role of fibroblast growth factor (Fgf) signaling in heart development and regeneration. Genetic manipulation studies by us and others have demonstrated that in the developing zebrafish embryo, Fgf signaling plays a critical role in axial patterning [12] and the induction and differentiation of heart progenitors during development [13,14,15,16]. The disruption of Fgf signaling leads to severe defects in cardiac progenitor specification [17], which is evident from the zebrafish *fgf8* mutant, *acerebellar* [14,18]. In contrast, increasing Fgf activity can expand cardiac progenitors during somitogenesis stages, and increase heart organ size [14,19]. To augment these studies, small molecules that modulate the activity of Fgf/Ras/Mapk signaling would permit reversible, transient, and temporally controlled pathway perturbation, which is difficult to achieve through genetic manipulations. Those small molecules could be useful to probe the mechanisms of heart growth during development, and potentially be applied to restoring cardiac function in congenital heart disease.

We previously performed a manual screen using a transgenic zebrafish line that responds to Fgf activity with expression of green fluorescent protein (GFP) in the mid-hindbrain boundary and trigeminal ganglia [20] to identify small molecules that hyperactivate Fgf/Ras/Mapk signaling [19]. We identified (*E*)-2-benzylidene-3-(cyclohexylamino)-2,3-dihydro-1*H*-inden-1-one (BCI), which hyperactivates Fgf signaling by inhibiting dual specificity phosphatase 6 (Dusp6), which is an Erk phosphatase, resulting in rapid Erk activation and the expression of Fgf target genes [19]. BCI is a specific small molecule chemical probe that is available to investigate the biology of Fgf signaling in development and regeneration, and it is selective for DUSP6 and DUSP1 over the related DUSP5 [19]. BCI also improves the regenerative capacity of the injured zebrafish heart [21,22]. Despite these specific biological activities, the BCI chemical structure contains an α,β-unsaturated ketone, which in theory could nonselectively modify cellular nucleophiles by Michael addition (although to date, no evidence exists that BCI has promiscuous effects) [23]. Therefore, we advanced our search for modifiers of Fgf signaling through a pilot screen of a chemical library of 1040 FDA-approved drugs with an automated imaging and analysis platform [19]. We identified two weak Fgf hyperactivators, pyrithione zinc and isoquinoline, which increased the expression of Fgf target genes and enhanced Fgf-dependent developmental markers [24]. While the pilot screen validated the zebrafish high-content assay, both agents have liabilities that impair their use as chemical probes for Fgf signaling. Isoquinoline is a promiscuous activator of stress signaling pathways [25], and pyrithione zinc is a topical antiseptic agent.

In order to further expand our small molecule toolbox to modulate Fgf signaling during development and regeneration, we have now screened the TimTec ActiProbe library. This library contains 10,000 discrete compounds, and importantly, contains clusters of structurally similar compounds to enable preliminary structure–activity relationship (SAR) studies during the primary screen. Through a carefully constructed decision network consisting of hit confirmation, cheminformatics filtering, evidence for SAR, whole organism toxicity evaluation, and orthogonal assays for Fgf pathway activity, we selected three compounds, namely ST006994, ST020101, and ST011282, for biological validation studies in zebrafish. Consistent with Fgf signaling hyperactivation, all three agents increased cardiac progenitor cells and cardiomyocyte numbers in the developing zebrafish heart. ST020101 and ST011282 activated Erk and were flagged by cheminformatics filters as potential covalent modifiers. In contrast, ST006994 hyperactivated the Fgf pathway without activating Erk, suggesting that its molecular target lies downstream of Erk. We posit that ST006994 could become a chemical probe, as it is the first agent that augments Fgf signaling without activating the ubiquitous Mapk signaling node, and thus potentially leading to reduced off-target side effects. Its unique mechanism of action could lead to the discovery of novel targets in zebrafish heart development and regeneration.

## 2. Results

### 2.1. High Throughput Screening and Hit Triage

**Figure 1:**
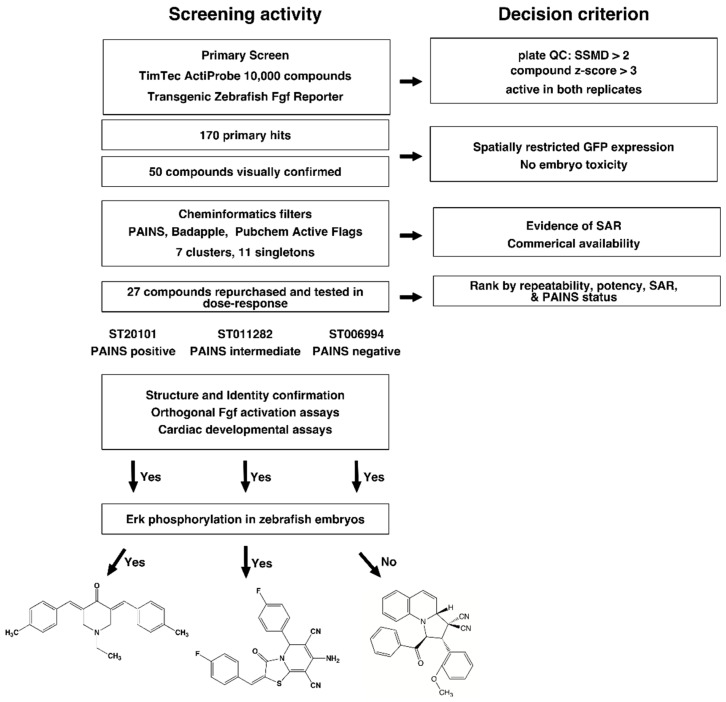
Whole organism high throughput, high content chemical screen for compounds that modulate fibroblast growth factor (Fgf)/Ras/Mapk signaling. Starting with a transgenic fluorescent zebrafish reporter line that reports on Fgf signaling, followed by a carefully constructed decision tree consisting of orthogonal assays, cheminformatics, and functional assays of heart development, the screen was designed to discover bona fide hyperactivators of Fgf signaling that affect embryonic heart development by various molecular mechanisms of action.

We followed a protocol that we developed and validated using a chemical library containing FDA-approved compounds [24] (Figure 1 depicts the overall discovery strategy). Using our previously described Cognition Network Technology (CNT) rule set [26], we interrogated the 10,000 member TimTec Actiprobe library in duplicate at a single concentration (30 µM) for compounds that increased GFP expression in the head. Duplicates were averaged, and plate performance was judged by strictly standardized mean difference (SSMD), as has been described by Zhang for siRNA screens [27], and was proven useful in prior zebrafish screens [7,24]. Plates that did not pass QC (SSMD < 2) were reanalyzed; the mean SSMD ± SD was 3.4 ± 1.5 (*n* = 125 plates). We identified 170 compounds that elevated GFP by more than three standard deviations above the plate mean (i.e., z-score > 3). Archived images were then examined visually to remove artifacts (precipitation, autofluorescence, death or high toxicity, analysis errors, or embryos that showed GFP expression in regions other than the head). The final list comprised 50 compounds that were subjected to a cheminformatics analysis for known or suspected assay interference, and reactive moieties and were binned into structural clusters.

### 2.2. Cheminformatics Analysis

Chemical similarity evaluation of the 50 primary hits identified seven clusters and 11 singletons. The largest cluster were enones (11 compounds), followed by imines (Schiff bases; eight compounds), malonodinitriles (six compounds), pyrrolidino-dihydroquinoline dinitriles (four compounds), and three smaller clusters (amino nitriles, sulfonamides and 2-amido thiophenes; three compounds each). The remaining hits appeared to be singletons. We then applied multiple cheminformatics filters to assess chemical liabilities (i.e., reactive moieties) and pan assay interference compounds (PAINS), namely ZINC15 (www.zinc15.docking.org), FAFDrugs4 (Free ADME-Tox Filtering Tool, http://fafdrugs3.mti.univ-paris-diderot.fr/), and False Positive Remover (www.cbligand.org). All algorithms flagged the enone cluster and some malonodinitriles as PAINS, based on potential electrophilic reactivity. The pyrrolidino-dihydroquinoline dinitriles and most singletons passed PAINS filters (Supplementary Table S1). Since at least the enones had previously been featured as “con artists” [28], we performed a predicted promiscuity search using Badapple (Bioactivity data associative promiscuity pattern learning engine, (http://pasilla.health.unm.edu/tomcat/badapple/badapple) [29]. Supplementary Table S2 documents the similarities and differences between PAINS and promiscuity analysis. For example, the enone cluster, as expected, showed evidence of promiscuity. The imines and many singletons, which had passed PAINS filters, also frequently hit in many assays. Conversely, the amino nitriles had low hit rates, despite being flagged as PAINS. The data were consistent with the assertion that PAINS compounds are not necessarily promiscuous [30,31], and that a carefully designed experimental testing regimen together with an awareness of potential liabilities is needed to advance the most promising therapeutic leads and/or informative probe compounds [32]. Therefore, we decided to analyze hits regardless of cheminformatics predictions. Twenty-seven compounds were commercially available, and a fresh sample was purchased for dose-response confirmation in the primary zebrafish assay, covering all of the clusters and eight out of the 11 singletons (Supplementary Table S1).

### 2.3. Hit Confirmation

**Figure 2:**
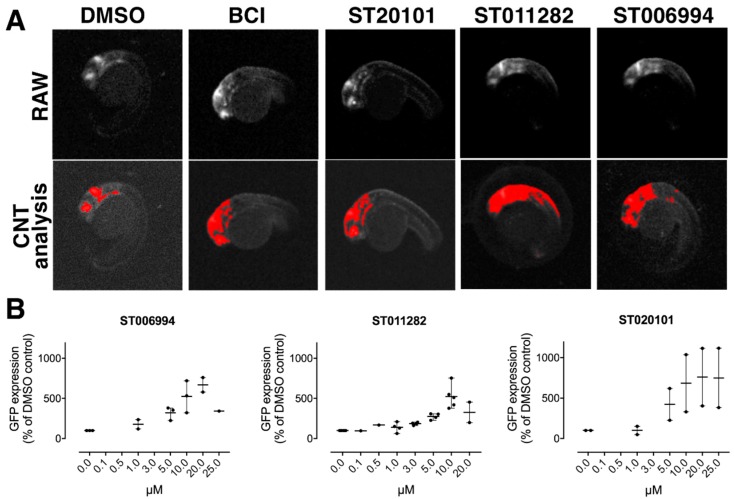
In vivo whole organism chemical screen identified new hyperactivators of Fgf/Ras/Mapk signaling. (**A**) Representative fluorescence micrographs of *Tg(dusp6:EGFP)* embryos (30 hpf) treated at 24 hpf for 6 h. Top panel, original scan image; bottom panel, images with Cognition Network Technology (CNT) algorithm applied. All of the compounds listed above images were at 10 µM. (**B**) Confirmatory dose response assay using repurchased small molecules: ST06994, ST011282, and ST020101. Each data point represents an independent assessment of total green fluorescent protein (GFP) intensity in the head of zebrafish larvae (*n* = 8). Horizontal bars denote the mean ± S.D. from two or three independent experiments.

A prescreen at three concentrations (20 µM, 40 µM, and 60 µM) confirmed activity in 20 compounds (Supplementary Figure S1), which were subsequently tested in five-point dose-response assays. Ten agents showed a measurable dose-response, although the magnitude of response for some was modest. The most robust activity was observed with compounds ST020087 and ST020101 from the enone cluster, the malonodinitriles ST001746 and ST030361, compound ST011282 (an amino nitrile), and the pyrrolidino-dihydroquinolines ST001193 and ST006694 (Figure 2 and Supplementary Figure S2). All of these compounds came from clusters with explicit SAR (i.e., the cluster contained inactive analogs), even though enones and malonodinitriles are electrophiles that have the potential to react nonselectively with sulfhydryl and amino groups. To further extend these preliminary SAR studies, we obtained a number of ST006994 analogs from the University of Pittsburgh Center for Chemical Methodologies and Library Development (UPCMLD) collection, some of which exhibited activity in zebrafish, whereas others did not (Supplementary Figure S3). In all of these studies, there was no correlation between Fgf hyperactivation, toxicity, and chemical reactivity, which was consistent with what we had observed previously with BCI analogs [33].

On the basis of these findings, we chose three compounds for follow-up studies: ST020101 as an example for a bona fide PAINS-positive compound to examine whether specific effects on heart development could be obtained with a known promiscuous agent; ST011282, which is without PAINS flags, but has potential chemical liabilities due to facile oxidation of the dihydropyridine substructure to the pyridinium ion, which then could act as a redox active electrophile, and ST006994, which had no discernible chemical or biological liabilities.

### 2.4. Confirmation of Fgf Hyperactivation by Orthogonal Assays

**Figure 3:**
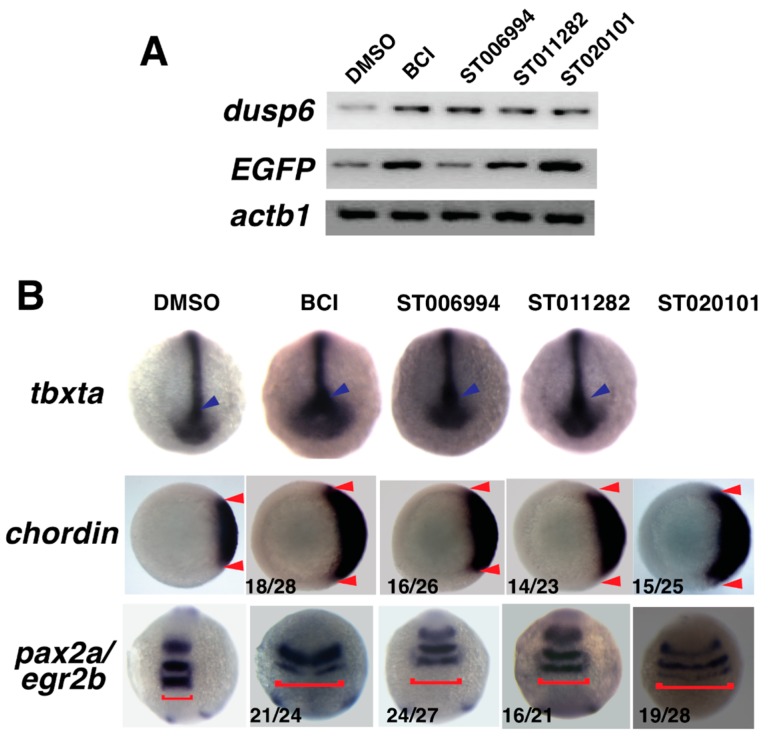
ST006994, ST020101, and ST011282 treatment induced the dorsalization and expansion of cardiac progenitor cells in zebrafish, which is a Fgf hyperactivation phenotype. (**A**) Semi-quantitative RT-PCR showing an increase of *dusp6* and *EGFP* transcripts after chemical treatment. (**B**) Representative images of whole mount in situ hybridization showing *tbxta, chordin*, and *pax2a/egr2b* expression after compound treatment. *tbxta* expression was increased in the tailbud (blue arrow), and *chordin* expression was expanded in the dorsal region when compared with DMSO control (distance between two red arrows). The neural markers *pax2a* and *egr2b* showed lateral expansion (red brackets), which is indicative of dorsalized phenotype from increased early Fgf activity.

In order to test if ST006994, ST011282, and ST020101 hyperactivated FGF signaling, we assayed for *dusp6* expression after compound treatment by semi-quantitative RT-PCR (Figure 3A). As a direct target of Fgf/Ras/Mapk signaling during the embryonic development of several species, *dusp6* is often used to indicate Fgf/Ras/Mapk activity [12,34]. *Tg(dusp6:EGFP)* embryos treated with ST006994, ST011282, and ST020101 for 3 h showed increased *dusp6* transcripts. Also, increased *EGFP* transcripts were noted in transgenic zebrafish, which correlated with increased EGFP fluorescence (Figure 3A) [19]. We treated embryos with drugs for 5 h from gastrulation (shield stage, 6 hpf), and analyzed the expression of the mesodermal gene *tbxta* (zebrafish *brachyury, previously known as ntla*) [35]. The expression of *tbxta* was greatly expanded within the notochord and the tailbud at the six-somite stage in ST006994 and ST011282-treated embryos (Figure 3B). Further, increasing the Fgf signal prior to gastrulation by either *dusp6* knockdown or by injecting *fgf8* mRNA [12,36] caused dorsalization, which includes the expansion of *chordin* expression [24]. In ST006994, ST011282, and ST020101-treated embryos, *chordin* was increased, which was indicative of Fgf hyperactivation (Figure 3B). Moreover, the treatment of shield stage embryos with ST006994, ST011282, or ST020101 expanded the expression of the neural markers *egr2b* (also known as *krox20*) *and pax2a*, which delineate the hindbrain rhombomeres 3 and 5 and the mid-hindbrain boundary, respectively (Figure 3B). These results were consistent with previous observations of Fgf hyperactivation [12,19,24]. Together, we confirmed that all three small molecules enhanced Fgf signaling in the zebrafish embryo, resulting in the increased transcription of several Fgf target genes.

### 2.5. Fgf Hyperactivators Influence Cardiac Chamber Development

**Figure 4:**
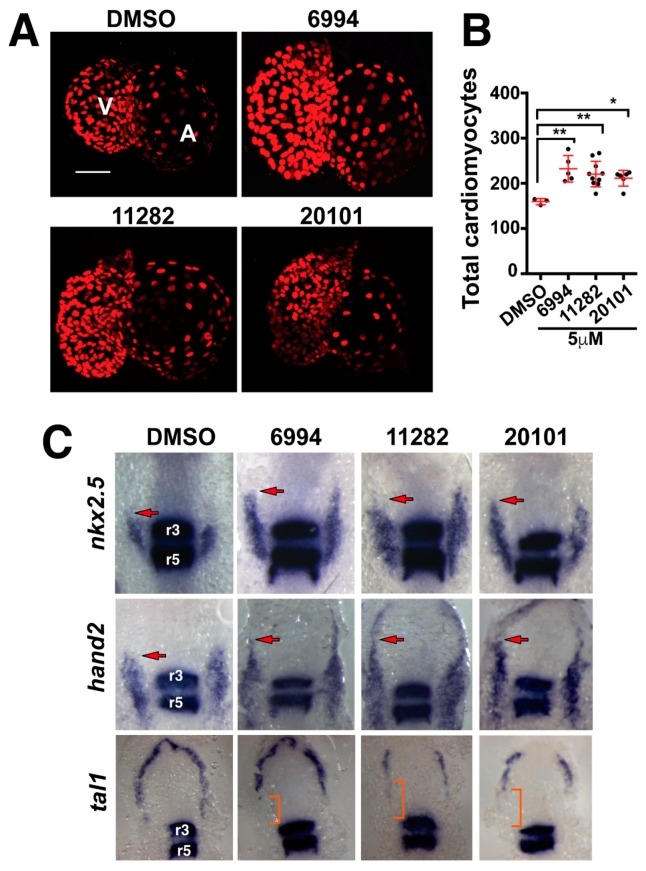
Fgf hyperactivators regulate heart size by increasing cardiac progenitor populations. (**A**) Fluorescence micrographs of cardiomyocyte nuclei from 72 hpf transgenic larvae treated for somite stages one through eight with 5 µM BCI, ST006994, ST011282, or ST020101. Images are representative for each compound. (**B**) The quantification of cardiomyocytes after small molecule treatment. All three compounds significantly increased the numbers of cardiomyocytes in the developing zebrafish heart. Each point represents a single larvae. (**C**) Whole mount in situ hybridization shows the expression of cardiac progenitor markers (*nkx2.5* and *hand2*) and endothelial progenitors (*tal1*) after compound treatment. *egr2b* was used to mark the hindbrain rhombomeres 3 and 5 (r3 and r5). Arrows mark the rostral most domain of the cardiac progenitor population. Brackets mark the region of decreased endothelial *tal1* expression.

During cardiac development, Fgfs play important roles in heart formation, particularly in defining organ size [14,16]. Consistent with these results, we had previously observed that BCI increased zebrafish cardiac progenitor cell pools and enlarged heart organ size upon treatment limited to somitogenesis stages [19]. We tested whether agents from this screen could also increase heart organ size by treating *Tg(5.7myl7:nDsRed2)^f^*^2^ embryos. This transgenic line expresses nuclear DsRed2 fluorescent protein under the control of a cardiac *myosin light chain 7* promoter, thus enabling the enumeration of differentiated cardiomyocytes [37]. We treated *Tg(5.7myl7:nDsRed2)^f^*^2^ embryos at the one-somite stage (10.3 hpf) with ST006994, ST011282, and ST020101, and at the eight-somite stage (11.3 hpf), compounds were washed out. At 72 hpf, treated transgenic embryos were scored for cardiomyocytes in the beating heart (Figure 4A). The enumeration of both ventricular and atrial cardiomyocytes revealed a statistically significant increase in nuclear DsRed2 expressing cells with all three compounds tested (Figure 4B). The observed increase in cardiomyocytes is consistent with an expansion of cardiac progenitor pools through Fgf hyperactivation at early somitogenesis stages, as the domains of expression of *nkx2.5* and *hand2*, two cardiac transcription factors, were expanded towards the rostral domain (Figure 4C), and a concomitant decrease was seen in the endothelial population. This indicates that the endothelial progenitor population undergoes a fate change to increase the cardiac population, as previously reported [38]. The data indicate that ST006994, ST011282, and ST020101 hyperactivated Fgf signaling, resulting in increased cardiac progenitor pools, which translated into increased numbers of ventricular cardiomyocytes and overall cardiac size.

### 2.6. ST006994 Activates Fgf Signaling without Hyperactivating Erk

**Figure 5:**
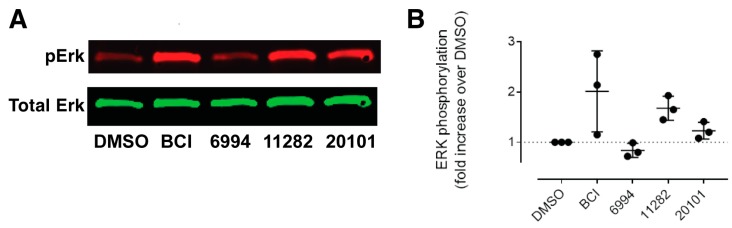
Erk phosphorylation status in compound treated embryos. (**A**) Representative Western blot showing the phosphorylation of Erk after a small molecule treatment of zebrafish embryos. (**B**) Quantification of pErk levels in the whole embryo lysates after compound treatment. Each data point represents the average pErk/Erk ratio from 50 pooled and lysed embryos. Horizontal bars denote the mean ± S.D. from three independent experiments.

During embryogenesis, Fgf ligands, upon binding to their cognate Fgf receptors, activate the Ras–Raf–Mapk cascade, resulting in the activation of Erk, which is a member of the Mapk family that regulates developmental processes, including the specification of cell fate and the promotion of cell survival [39]. BCI, the current standard chemical probe for the enhancement of Fgf/Ras/Mapk signaling, increases the phosphorylation of Erk through the inhibition of Dusp6; however, initial experiments showed that none of the agents identified in the large scale screen inhibited DUSP6 in a chemical complementation assay [40] (Supplementary Figure S4 and data not shown). ST011282 and ST020101, similar to BCI, increased Erk phosphorylation, whereas ST006994 did not (Figure 5). Since the *Dusp6* promoter has been characterized as a direct target of Ras/Mapk signaling [20,41], our findings suggest that ST006994 is acting to enhance Fgf target gene transcription downstream of Erk.

## 3. Discussion

In this report, we extended our previously developed automated zebrafish high-content screening platform to interrogate a discovery library of 10,000 compounds. The goal of this screen was to interrogate a larger chemical space to identify new Fgf hyperactivating agents that specifically activate Fgf/Ras/Mapk pathways while lacking chemical reactivity and/or biological promiscuity. We developed a secondary assay paradigm that incorporated multilevel decision gates, including secondary assays for Fgf target gene expression and cardiomyocyte expansion, to biologically validate newly identified small molecules. Cheminformatics tools were used for structure similarity clustering and structure–activity relationships to identify potential liabilities that impair probe development and generate hypotheses relating chemical structure to biological activity.

### 3.1. Unique Features of the TimTec ActiProbe Library

The TimTec Actiprobe library that was used in this study is a collection of highly diverse synthetic molecules arranged by Jarvis Patrick clustering [42] (http://www.timtec.net/actiprobe-10k-library.html), where small molecules with structural similar fingerprints are arranged in nearby plate wells. The remaining wells are being filled with chemically unique singletons. This design permits a preliminary assessment of structure–activity relationships during the primary screen.

### 3.2. High-Content Screening in Transgenic Fluorescent Zebrafish

We used our previously described automated screening platform [24,26] to identify compounds that induced Fgf activity as measured by a fluorescent biosensor [20]. The screen was conducted according to accepted HTS criteria, with plates containing positive and negative controls to monitor in screen assay performance. The primary QC criterion was the strictly standardized mean difference (SSMD), as we and others had previously found useful for zebrafish screening [7,24]. The primary screen identified 50 hits after visual inspection of archived scan images for toxicity and GFP expression in areas other than the head. Cheminformatics clustering revealed seven clusters and 11 singletons (Supplementary Data S1). Some clusters showed evidence of explicit SAR, illustrating the utility of the TimTec library design. These included the enones, pyrrolidino-dihydroquinoline dinitriles, and amino nitriles. Dose-response studies further confirmed SAR in these clusters, and one representative from each of the three clusters was chosen for follow-up validation studies.

### 3.3. Hypotheses Derived from Cheminformatics

We used a variety of cheminformatics tools to assess potential liabilities and promiscuities (i.e., filters for pan assay interference compounds (PAINS) filters). Although most PAINS filtering algorithms are based on the same 480 structural elements that were discovered in the initial PAINS publication [43], the results delivered by different analysis engines were not uniform. In particular, ZINC15 did not appear to apply as stringent criteria as other analysis engines (Supplementary Data S1). A comparison between PAINS filters and promiscuity analysis based on actual assay data (using Badapple) revealed some similarities (i.e., the enones and malonodinitriles were flagged as PAINS and showed promiscuous activity), but also some notable discrepancies, as all of the imines, which had uniformly passed PAINS filters, were frequent hitters (Supplementary Data S2). This may be in part because Badapple retrieves assay hit rates based on substructures, which can be problematic, since substructure filters do not consider activating or deactivating substituents, or steric constraints. Conversely, some of the amino nitriles, which had been flagged as PAINS, had very good Badapple scores, indicating low hit rates despite their chemical reactivity (Supplementary Data S2). The findings were consistent with a recent analysis of large scale datasets showing that most PAINS compounds have very low hit rates [30,31]. They also support the paradigm that not all compounds that are chemically reactive are necessarily promiscuous (i.e., are not PAINS in a literal sense) [30,31], but that reactive structures are generally undesirable unless they are target-specific or reversible covalent modifiers [28,32,43]. A large body of literature now documents that covalent inhibitors can be rationally designed [44,45] and multiple drugs on the market are covalent modifiers (e.g., penicillin, omeprazole, clopidogrel) [46]. We therefore decided to examine compounds regardless of whether they had been flagged as PAINS, while being mindful of potential chemical liabilities. The final three lead agents that were chosen for functional studies belonged to three classes: one bona fide PAINS compound (ST020101) [28], a non-PAINS compound with predicted chemical reactivity but low Badapple score (ST011282), and an agent that passed all filters (ST006994). This was done to test the following hypotheses: (1) whether reactive and promiscuous agents could still elicit specific biological effects; (2) whether chemical reactivity correlated with biological activity; and (3) whether compounds from the different classes would differ in their mechanism of action.

### 3.4. Zebrafish Chemical Screening Identified a Non-Canonical Hyperactivator of Fgf Signaling

All three lead compounds induced Fgf target genes in the developing zebrafish embryo and caused a fate change from endothelial cell progenitors to cardiac progenitors that resulted in increased numbers of cardiomyocytes in the zebrafish larval heart. Thus, chemical reactivity did not affect biological activity. We then explored possible mechanisms for Fgf hyperactivation, and found that the two electrophiles (ST020101 and ST011282) caused Erk phosphorylation, similarly to BCI. Interestingly, ST006994 did not enhance Erk phosphorylation, indicating that it augments rather than stimulates Erk activity, possibly by affecting (a) target(s) downstream of Mapk.

In summary, we have designed and executed a high-throughput phenotypic in vivo chemical screen in transgenic zebrafish to identify new agents that modulate Fgf/Ras/Mapk signaling. The decision network included cheminformatics, structure–activity relationship studies, secondary phenotypic assays of heart development, and mechanism of action studies within the Mapk pathway. The results show that specific effects on zebrafish heart development can be achieved by multiple mechanisms along the Fgf/Ras/Mapk cascade. Two agents (ST020101 and ST011282) hyperactivated Fgf signaling by activating Erk, similar to what we had previously observed with the gold standard, BCI. However, in contrast to BCI, activation of Erk by ST020101 and ST011282 did not involve the inhibition of Dusp6 or Dusp1 (data not shown). The mechanism by which ST020101 and ST011282 activate Erk is unknown, but because of their chemical reactivity, could be secondary to a general stress response. Notwithstanding, both agents were devoid of developmental toxicity at concentrations that hyperactivated Fgf signaling and enlarged cardiomyocyte progenitor pools, indicating that PAINS-like and promiscuous agents can elicit specific targeted responses. However, most interesting was the discovery of ST006994, which appears to hyperactivate Fgf signaling by a molecular mechanism that does not involve Erk activation. The biological consequences of this finding are currently not known, but it is tempting to speculate that ST006994 could be a more specific chemical probe for Fgf-mediated heart development than existing agents, and could have fewer side effects because it bypasses the ubiquitous Mapk signaling node. The mechanism by which ST006994 hyperactivates Fgf signaling could also lead to the discovery of novel targets in the Fgf/Ras/Mapk pathway.

## 4. Materials and Methods

### 4.1. Zebrafish Handling and Maintenance

All of the experiments using zebrafish were carried out with prior review and approval by the University of Pittsburgh Institutional Animal Care and Use Committee. Heterozygous *Tg(dusp6:EGFP)*^*pt*6^ embryos (24 hpf) were obtained by single pair homozygous out crossing [20].

### 4.2. Plate Preparation and Processing

The TimTec Actiprobe library collection of 10,000 compounds was screened at a final concentration of 30 µM in 1% DMSO. Each well of a 96-well round bottom plate (TTP Labtech) was loaded with a single 24-hpf transgenic embryo in 200 µL of E3 (5 mM NaCl, 0.17 mM KCl, 0.33 mM CaCl_2_, 0.33 mM MgSO_4_). Embryos were stage matched from a single homozygous outcross mating pair; only embryos that were viable and showed transgene expression were used. 100× concentrated drug stocks in DMSO were dispensed into each well, and the plate was briefly shaken to ensure proper mixing. Each microplate contained eight wells of vehicle controls (1% DMSO) and eight wells of 10 µM of BCI as the positive control. Duplicate plates were run to eliminate loading and toxicity artifacts, and to limit variability. Plates were covered with gas-permeable plate seals and incubated at 28.5 °C for 5 h before imaging.

### 4.3. Automated Embryo Imaging and Analysis

Embryos were anesthetized with MS222 (0.61 mM tricaine methanesulfonate, Sigma-Aldrich, St. Louis, MO, USA) at the end of the 5-h drug treatment to restrict their movement during imaging. Images were acquired on an ImageXpress Ultra high-content reader (Molecular Devices, Sunnyvale, CA, USA) using a 4× objective at a fully open pinhole size and excitation/emission wavelengths of 488/525 nm (GFP) [26]. Archived fluorescence micrographs were uploaded into Definiens Developer (Definiens AG, Munich, Germany) using the Cellenger module, and analyzed for GFP expression in the head using a slightly modified version of our previously described CNT algorithm [26]. The algorithm was designed to identify the zebrafish larvae, irrespective of their orientation in the well. A GFP threshold was defined based on overall larval fluorescence. Regions within the zebrafish larva were classified as positive for GFP expression if their fluorescence intensity exceeded the threshold GFP intensity. Total head structures’ brightness was defined as the integrated GFP intensity of the four brightest head structures. Embryos exhibiting head structures brightness that was at least three-fold greater than the standard deviation (SD) from the mean of all of the samples on the microplate excluding controls (z-score >3), and that repeated on both plates, were considered hits. Plate performance was judged by strictly standardized mean difference (SSMD) between positive and negative controls, as described by Zhang [27]; plates with SSMDs <2 were rescreened.

### 4.4. Cheminformatics Analysis

Hits from the primary screen were subjected to cheminformatics filters and clustered based on structural similarity. Four independent databases/tools were used, all of them incorporating a set of 480 structural features found in compounds that had shown activity in multiple alpha screens (subsequently termed PAINS, or pan assay interference compounds) [43]. Specifically, we queried ZINC15 (http://zinc15.docking.org), FAFDrugs4 (Free ADME-Tox Filtering Tool, http://fafdrugs3.mti.univ-paris-diderot.fr/), False Positive Remover (www.cbligand.org), and Badapple (Bioactivity data associative promiscuity pattern learning engine, http://pasilla.health.unm.edu/tomcat/badapple/badapple) [29]. Compounds were then grouped by chemical similarity by an experienced organic chemist.

### 4.5. Dose-Response Confirmation and Analysis of Compound Identity and Purity

Dose-response studies were carried out on 27 agents with fresh repurchased samples (TimTec) using eight embryos per condition. Compounds were first tested in a prescreen at 20 µM, 40 µM, and 60 µM; compounds that showed activity in the prescreen at any concentration were retested in five-point concentration curves to determine the concentration required to elicit a half-maximal response (EC50). The final three candidates were repurchased again in 10-mg quantities. ST006994 analogs from the UPCMLD library were retrieved from solid archived stocks. Identity and purity were ascertained by high-resolution mass spectrometry (HRMS) and electrophoretic light scattering (ELS) to confirm purity and identity (Supplementary Data S3 and S4). HPLC data were obtained on a Thermo Scientific Accela HPLC system using 3-μL injections on a 2.1 × 50 mm 3.5 µm Waters XTerra C_18_ column eluting with MeCN/H_2_O/MeOH containing 0.1% formic acid (flow rate of 500 μL/min from 3:92:5 at 0–0.5 min to 93:2:5 at 4.0 min, back to 3:92:5 from 6.0 to 7.5 min). Absorbance was monitored at 210 nm, 220 nm, and 254 nm. HRMS data were obtained on a Thermo Scientific Exactive HRMS (ESI-). Samples were dissolved in MeCN, and 5-μL aliquots were direct-injected into the probe head (−60 V, 350 °C). Mass accuracy was determined with Thermo Xcalibur software. Dose-response curves for the final three compounds were repeated at least two times; data from all of the independent experiments were averaged and analyzed by a four parameter logistic equation in GraphPad Prism. 

### 4.6. Whole Mount In Situ Hybridization

Whole mount in situ protocols were carried out as described previously [47]. The following probes generated in previous studies were used in this study: *dusp6* [12], *chordin* [48], *egr2b* [49], *pax2a* [50], *nkx2.5*, *hand2*, and *tal1* [19]. Embryos were treated with compounds at 3 hpf until shield stage (6 hpf) and fixed in 4% paraformaldehyde for *tbxta* and *chordin* staining, respectively. For *nkx2.5*, *hand2*, and *tal1* staining, embryos were treated with compounds at the one-somite stage and fixed at the 10-somite stage (~13.5 hpf). *egr2b* staining was used to demarcate the hindbrain.

### 4.7. Cardiomyocyte Imaging

To quantify numbers of cardiomyocytes in zebrafish hearts at 72 hpf, *Tg(5.7myl7:nDsRed2)^f^*^2^ embryos were used [37]. Embryos were incubated at 28.5 °C and treated with DMSO, ST006994, ST011282, or ST020101 at the one-somite stage [51] until embryos reached the eight-somite stage. Drugs were washed off using E3, and embryos were incubated at 28.5 °C until 72 hpf. Prior to imaging, embryos were anesthetized with MS222 (tricaine methane sulfonate) and mounted in custom-made plexiglass molds. Live imaging was carried out using an upright Olympus Fluoview FV1000 MPE Multiphoton confocal microscope with a 25× objective. Sequential confocal images were captured using the 750-nm laser line with a standardized step size of 3 μm in the z-direction. Three-dimensional (3D) reconstructions of confocal stacks were made using Imaris 7.2 X64 software (BITPLANE Scientific, Switzerland). The quantification of red-labeled cardiomyocyte nuclei was performed interactively on the 3D reconstruction image. Objects were classified using Imaris software as DsRed2 positive if they exceeded an interactively determined radius (4.9 µm) and their average intensity exceeded a threshold based on background fluorescence. The experiment was repeated twice with similar results.

### 4.8. Western Blotting

Zebrafish embryos (24 hpf) after treating with drugs for 30 min were dechorionated and subjected to gentle pipetting to remove yolks in ice-cold PBS with 1:10 (wt/vol) of protease inhibitors (Complete Mini, EDTA-free, Roche#11836170001, Mannheim, Germany) and phosphatase inhibitor (Phos STOP Roche#04906837001). The embryos were centrifuged at 3000 G for 5 min. After removing PBS, deyolked embryos were lysed in Tris buffer (20 mM of Tris-HCl pH 7.5, 150 mM of NaCl, 1 mM of EDTA, 1 mM of EGTA, 1% Triton X) with 1:10 (wt/vol) of protease and phosphatase inhibitors. Equal amounts of protein (50 ug) were heat denatured and separated on 10% SDS-PAGE and transferred to nitrocellulose membranes using a semidry blot system (BIORAD). Primary antibodies used were: mouse anti phospho-ERK (Sigma#M8159) (1:500) and rabbit anti-ERK (Cell signaling #4695S) (1:1000). Secondary antibodies were diluted 1:15,000 and were: IRDye 800CW donkey anti-rabbit (Licor, 926-32213) and IRDye 680 goat anti mouse (Odyssey, 926-68020). Antibodies were diluted in Odyssey blocking buffer containing 0.2% Tween 20 (National diagnostics). Blots were scanned using a Li-cor Odyssey CLx Infrared imaging system, and band intensities were quantified and normalized using Image Studio software (Li-cor, Lincoln, NE, USA).

## Supplementary Materials

The following are available online at, Figure S1: Three-point dose response pre-screen on 27 repurchased primary confirmed hits. Figure S2: Dose-response curves for compounds that were not selected for follow-up studies. Figure S3: SAR studies of ST006994 analogs from the UPCMLD library. Figure S4: Representative Chemical Complementation assay shows new compounds do not inhibit DUSP6. Data S1: PAINS analysis of primary confirmed hits. Data S2: Promiscuity analysis of primary confirmed hits. Data S3: LC-HRMS data for ST020101, ST011282, and ST00694. Data S4: Structure/identity confirmation and LC-HRMS of ST006994 analogs from the UPCMLD library.

## Abbreviations

BCI	(E)-2-benzylidene-3-(cyclohexylamino)-2,3-dihydro-1H-inden-1-one
Dusp	dual specificity phosphatase
Erk	extracellular signal related kinase
Fgf	fibroblast growth factor
Mapk	mitogen activated protein kinase
SAR	structure-activity relationship
