# Supplementary material for: A High-Content Screen Reveals New Small-Molecule Enhancers of Ras/Mapk Signaling as Probes for Zebrafish Heart Development
Source: Molecules. 2018 Jul 11;23(7):1691. doi: 10.3390/molecules23071691 (PMC6099644; doi:10.3390/molecules23071691)
Supplement: Supplementary file 1 [file molecules-23-01691-s001.zip › molecules-327113 Supplementary Figures & Data/Supplementary Data Legends.pdf]

A high-content screen reveals new small-molecule enhancers of Ras/Mapk signaling as probes for zebrafish heart development.

Manush Saydmohammed[1], Laura L. Vollmer[2], Ezenwa O. Onuoha[1], Taber S. Maskrey[4], Gregory Gibson[3], Simon C. Watkins[3], Peter Wipf[4], Andreas Vogt[2, 5], Michael Tsang[1]

[1]Department of Developmental Biology, University of Pittsburgh, BST3, 3501 5th Avenue, Pittsburgh, PA 15213

[2]The University of Pittsburgh Drug Discovery Institute, 200 Lothrop Street, Pittsburgh, PA 15260

[3]Department of Cell Biology, University of Pittsburgh, 3500 Terrace Street, Pittsburgh, PA 15213

[4]Department of Chemistry, 219 University Drive, University of Pittsburgh, Pittsburgh, PA 15260

[5]Department of Computational and Systems Biology, University of Pittsburgh, Pittsburgh, PA 15213

Address correspondence to MT (tsang@pitt.edu) or AV (avogt@pitt.edu).

Supplementary Data Legends:

Supplementary Data 1: PAINS analysis of primary confirmed hits. Fifty visually confirmed primary screen hits were analyzed for predicted pan assay interference compounds (PAINS) by publicly available analysis engines (ZINC15, www.zinc15.docking.org); FAFDrugs4 (Free ADME-Tox Filtering Tool, http://fafdrugs3.mti.univ-paris-diderot.fr/); False Positive Remover (www.cbligand.org); accessed May 2018). Compounds were grouped by structural similarity. The last

column (cluster coverage) denotes the number of commercially available compounds that were repurchased from each cluster.

Supplementary Data 2. Promiscuity analysis of primary confirmed hits.
Fifty visually confirmed primary screen hits were analyzed for predicted promiscuity using the Bioactivity data associative promiscuity pattern learning engine (Badapple); http://pasilla.health.unm.edu/tomcat/badapple/badapple; accessed June 2018). The scoring scheme is as follows: green, pScore 0-100, no indication of promiscuity; yellow, 100-300; weak indication of promiscuity; red, >300, strong indication of promiscuity; no color, unknown; no data. For each molecule, the highest scoring scaffold determines overall score.

Table headings are: mon_smi, compound structures; scaf_smi, scaffold structures; pScore, Badapple assigned promiscuity estimate (see scoring scheme above); Substances Tested, number of compounds containing scaffold substructure; Samples Tested, number of times substructure was tested; Assays Active, number of assays in which substructure was active; Assays Tested, number of assays substructure was tested; Percent Assays Active, calculated ratio of Assays Active/Assays Tested *100.

Supplementary Data 3. LC-HRMS data for ST020101, ST011282, and ST00694.

Supplementary Data 4. Structure/identity confirmation and LC-HRMS of ST006994 analogs from the UPCMLD library. For technical details please refer to the Materials and Methods Section.
