# Supplementary material for: A High-Content Screen Reveals New Small-Molecule Enhancers of Ras/Mapk Signaling as Probes for Zebrafish Heart Development
Source: Molecules. 2018 Jul 11;23(7):1691. doi: 10.3390/molecules23071691 (PMC6099644; doi:10.3390/molecules23071691)
Supplement: Supplementary file 1 [file molecules-23-01691-s001.zip › molecules-327113 Supplementary Figures & Data/Supplementary Figure Legends.pdf]

A high-content screen reveals new small-molecule enhancers of Ras/Mapk signaling as probes for zebrafish heart development.

Manush Saydmohammed[1], Laura L. Vollmer[2], Ezenwa O. Onuoha[1], Taber S. Maskrey[4], Gregory Gibson[3], Simon C. Watkins[3], Peter Wipf[4], Andreas Vogt[2, 5], Michael Tsang[1]

[1]Department of Developmental Biology, University of Pittsburgh, BST3, 3501 5th Avenue, Pittsburgh, PA 15213

[2]The University of Pittsburgh Drug Discovery Institute, 200 Lothrop Street, Pittsburgh, PA 15260

[3]Department of Cell Biology, University of Pittsburgh, 3500 Terrace Street, Pittsburgh, PA 15213

[4]Department of Chemistry, 219 University Drive, University of Pittsburgh, Pittsburgh, PA 15260

[5]Department of Computational and Systems Biology, University of Pittsburgh, Pittsburgh, PA 15213

Address correspondence to MT (tsang@pitt.edu) or AV (avogt@pitt.edu).

Supplementary Figure Legends

Supplementary Figure S1: Three-point dose response pre-screen on 27 repurchased primary confirmed hits.

Supplementary Figure S2: Dose-response curves for compounds that were not selected for follow-up studies.

Supplementary Figure S3: SAR studies of ST006994 analogs from the UPCMLD library.

Supplementary Figure S4: Representative Chemical Complementation assay shows new compounds do not inhibit DUSP6. Data are the averages +/- SEM. BCI, n=4; ST006994, n=3 (repurchased materials that confirmed in LC-MS). For ST006994 errors are smaller than the symbols. ST011282 n=1.
