# Supplementary material for: A High-Content Screen Reveals New Small-Molecule Enhancers of Ras/Mapk Signaling as Probes for Zebrafish Heart Development
Source: Molecules. 2018 Jul 11;23(7):1691. doi: 10.3390/molecules23071691 (PMC6099644; doi:10.3390/molecules23071691)

Supplementary Figure 3. 6994 analogs from the UPCMLD library

Active at 10 and 20 μM

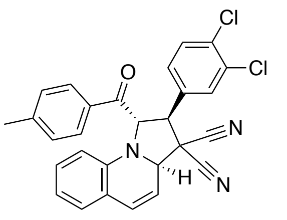

UPCMLDCBRC1000784

Chemical Formula: $C_{28}H_{19}Cl_2N_3O$
Molecular Weight: 484.38

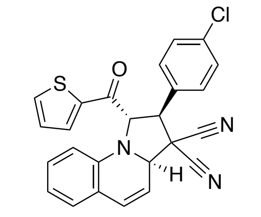

UPCMLDCBRC1000802

Chemical Formula: $C_{25}H_{16}ClN_3OS$
Molecular Weight: 441.93

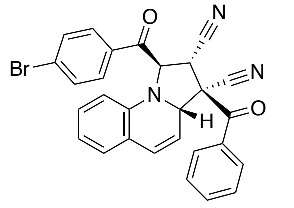

UPCMLDCBRC1001299

Chemical Formula: $C_{28}H_{18}BrN_3O_2$
Molecular Weight: 508.38

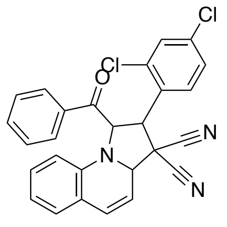

UPCMLDCBRC1033229

Chemical Formula: $C_{27}H_{17}Cl_2N_3O$
Molecular Weight: 470.35

Inactive at 10 and 20 μM

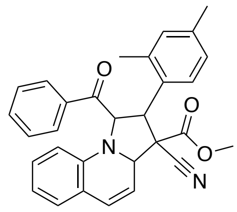

UPCMLDCBRC1002602

Chemical Formula: $C_{30}H_{26}N_2O_3$
Molecular Weight: 462.55

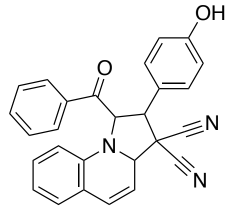

UPCMLDCBRC1004885

Chemical Formula: $C_{27}H_{19}N_3O_2$
Molecular Weight: 417.47

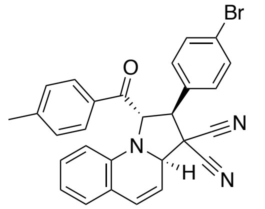

UPCMLDCBRC1038747

Chemical Formula: $C_{28}H_{20}BrN_3O$
Molecular Weight: 494.39

Autofluorescent

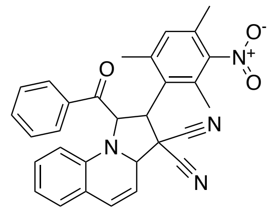

UPCMLDCBRC1033038

Chemical Formula: $C_{30}H_{24}N_4O_3$
Molecular Weight: 488.55

HPLC and HRMS Data:

HPLC data were obtained on a Thermo Scientific Accela HPLC system using 3 µL injections on a 2.1 x 50 mm 3.5 µm Waters XTerra C_{18} column eluting with MeCN/H_2O/MeOH containing 0.1% formic acid (flow rate of 500 µL/min from 3:92:5 at 0-0.5 min to 93:2:5 at 4.0 min, back to 3:92:5 from 6.0 to 7.5 min). Absorbance was monitored at 210, 220, and 254 nm. HRMS data were obtained on a Thermo Scientific Exactive HRMS (ESI-). Samples were dissolved in MeCN and 5 µL aliquots were direct-injected into the probe head (-60 V, 350 °C). Mass accuracy was determined with Thermo Xcalibur software.

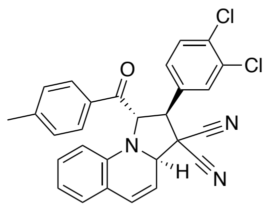

UPCMLDCBRC1000784. HPLC retention time 6.43 min. HRMS (HESI) *m/z* calcd for $C_{28}H_{18}N_3O_2Cl_2$ $[M-H]^-$ 482.0821, found 482.0841.

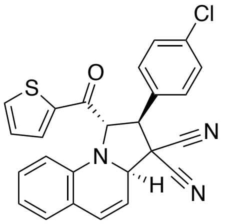

UPCMLDCBRC1000802. HPLC retention time 5.16 min. HRMS (HESI) *m/z* calcd for $C_{25}H_{15}N_3OClS$ $[M-H]^-$ 440.0619, found 440.0637.

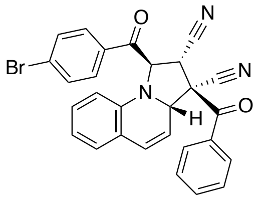

UPCMLDCBRC1001299. HPLC retention time 5.89 min. HRMS (HESI) *m/z* calcd for $C_{28}H_{17}N_3O_2Br$ $[M-H]^-$ 506.0499, found 506.0526.

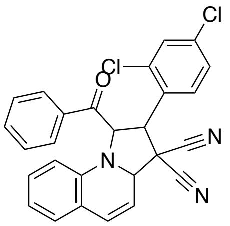

UPCMLDCBRC1033229. HPLC retention time 5.46 min. HRMS (HESI) *m/z* calcd for $C_{27}H_{16}N_3OCl_2$ $[M-H]^-$ 468.0665, found 468.0684.

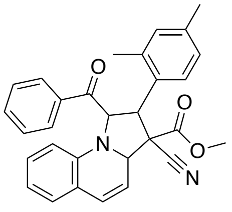

UPCMLDCBRC1002602. HPLC retention time 5.81 min. HRMS (HESI) *m/z* calcd for $C_{30}H_{25}N_2O_3$ $[M-H]^-$ 461.1860, found 461.1872.

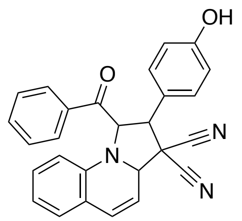

UPCMLDCBRC1004885. HPLC retention time 4.90 min. HRMS (HESI) *m/z* calcd for $C_{27}H_{18}N_3O_2$ $[M-H]^-$ 416.1394, found 416.1408.

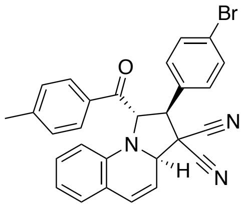

UPCMLDCBRC1038747. HPLC retention time 5.45 min. HRMS (HESI) *m/z* calcd for $C_{28}H_{19}N_3OBr$ $[M-H]^-$ 492.0706, found 492.0724.

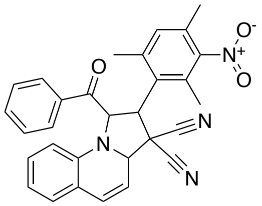

UPCMLDCBRC1033038. HPLC retention time 5.30 min. HRMS (HESI) *m/z* calcd for $C_{30}H_{23}N_4O_3$ $[M-H]^-$ 487.1765, found 487.1778.

D:\data\Pete\C1000784_1
C:\Xcalibur\methods\walkup\10min_0395A05M_pn.meth

6/4/2018 11:09:02 AM

UPCMLDCBRC1000784

RT: 0.00 - 9.98

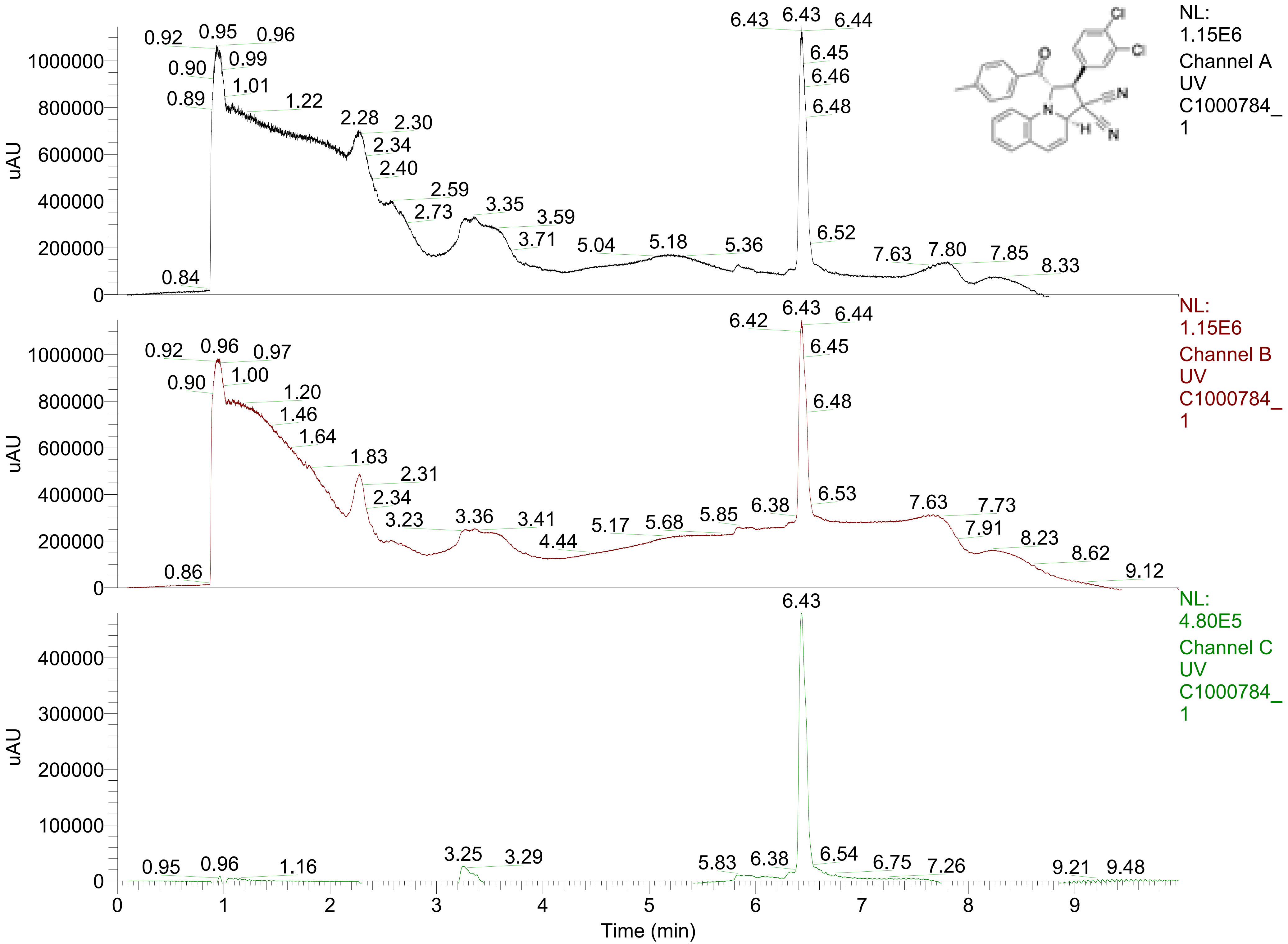

RT: 0.00 - 0.73

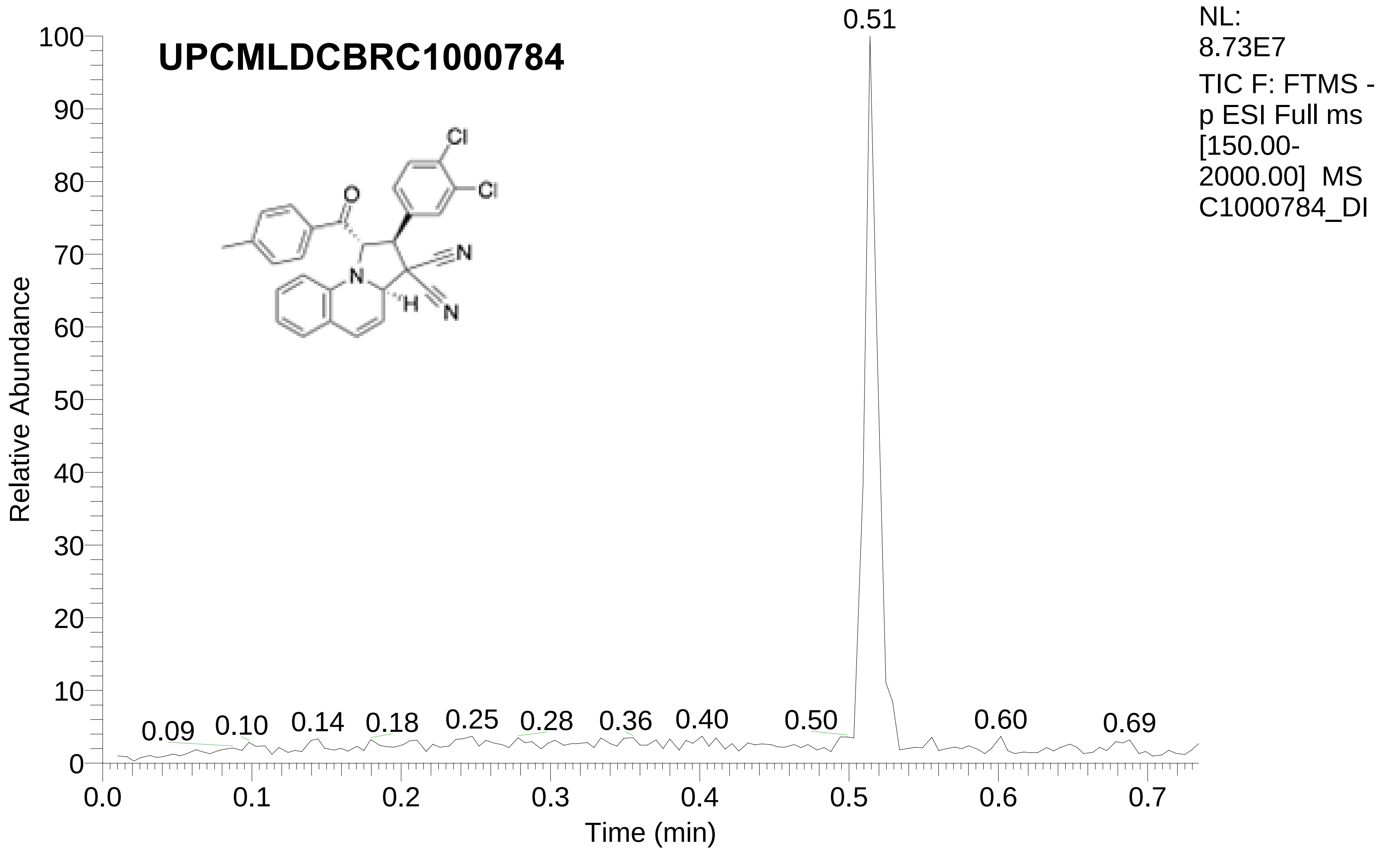

C1000784_DI #98-101 RT: 0.51-0.52 AV: 4 NL: 6.70E5
T: FTMS - p ESI Full ms [150.00-2000.00]

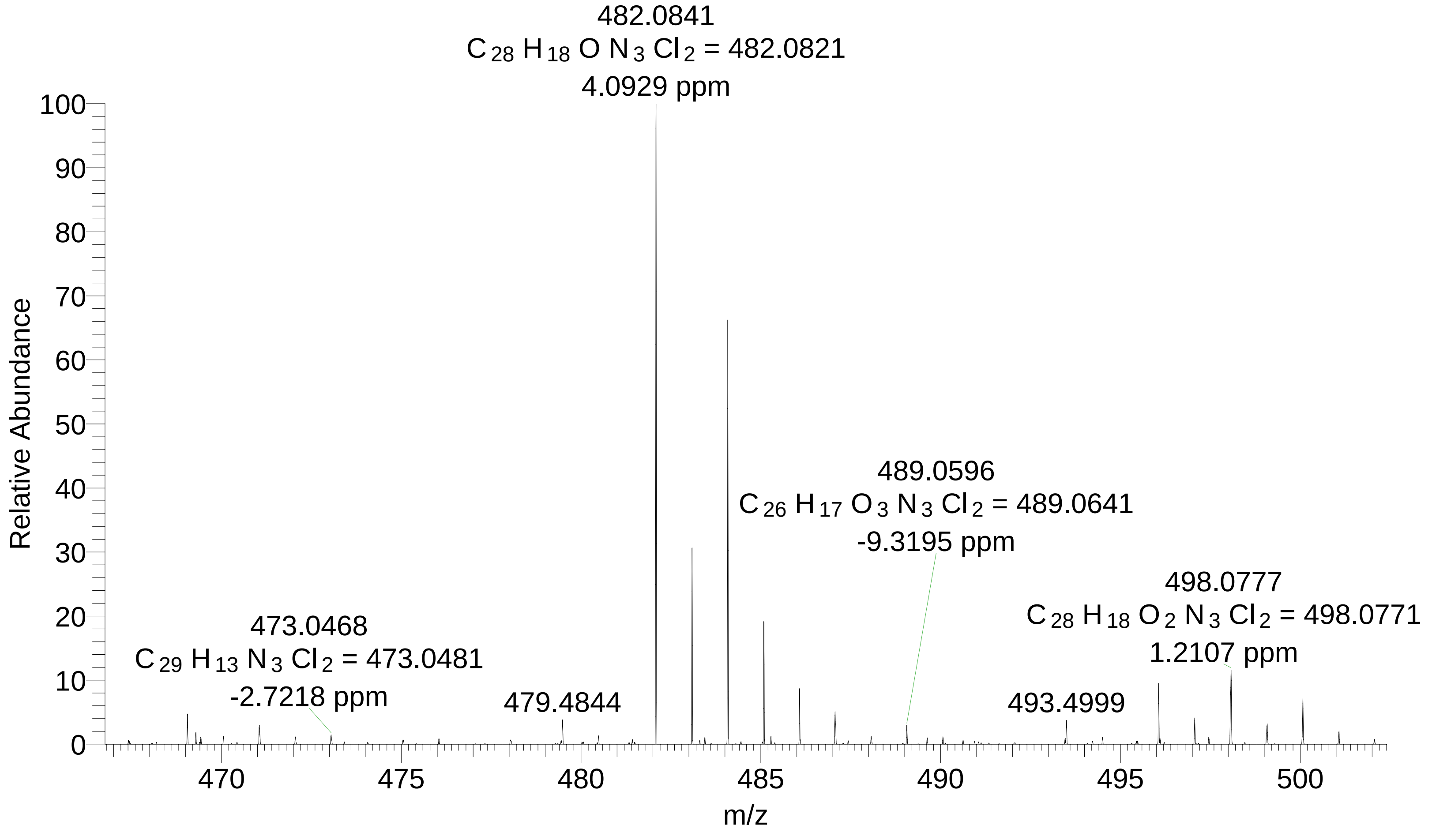

D:\data\Pete\C1000802_1_180605095823 6/5/2018 9:58:23 AM **UPCMLDCBRC1000802**
C:\Xcalibur\methods\walkup\10min_0395A05M_pn.meth

RT: 0.00 - 9.98

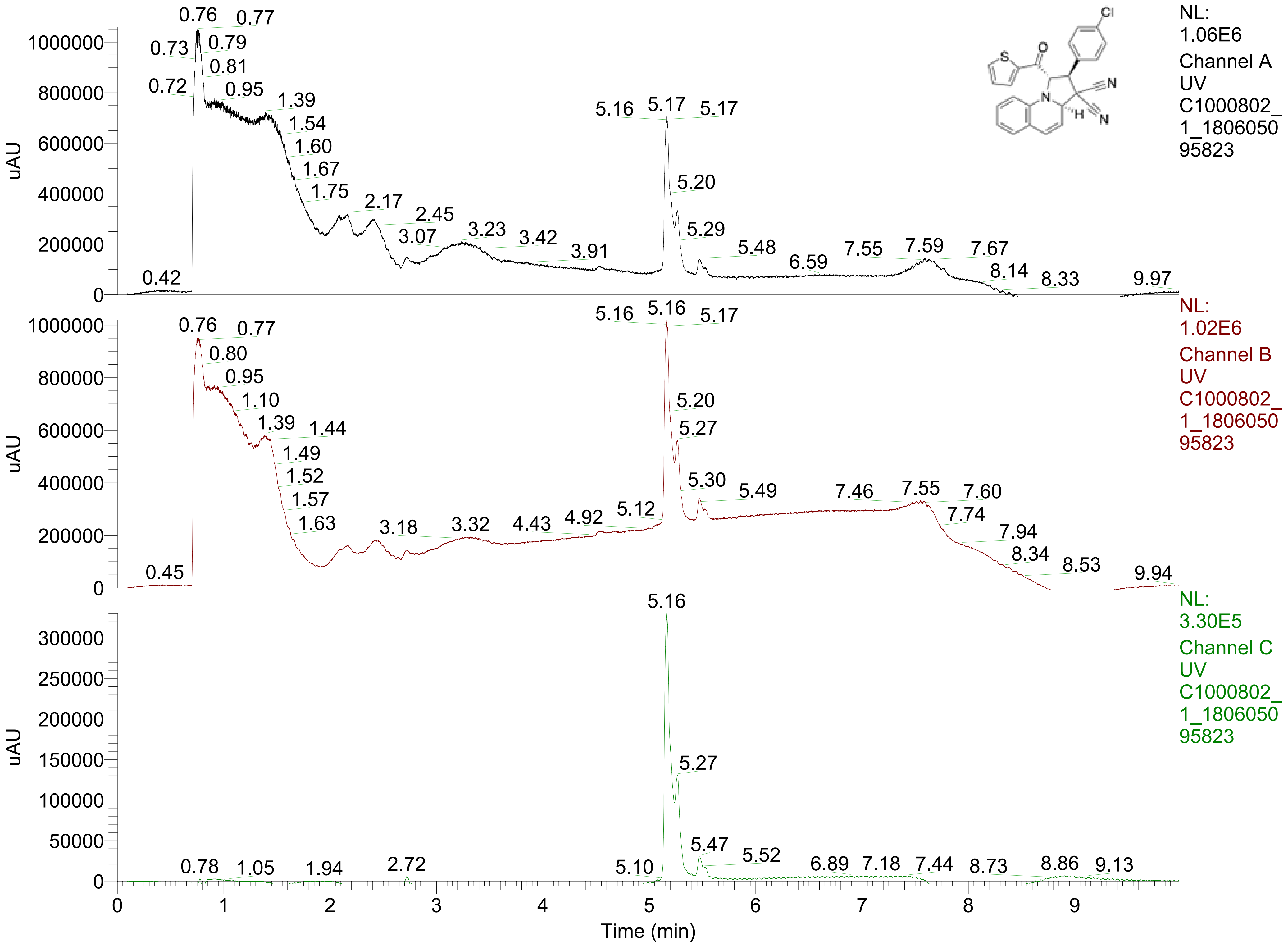

RT: 0.70 - 0.91

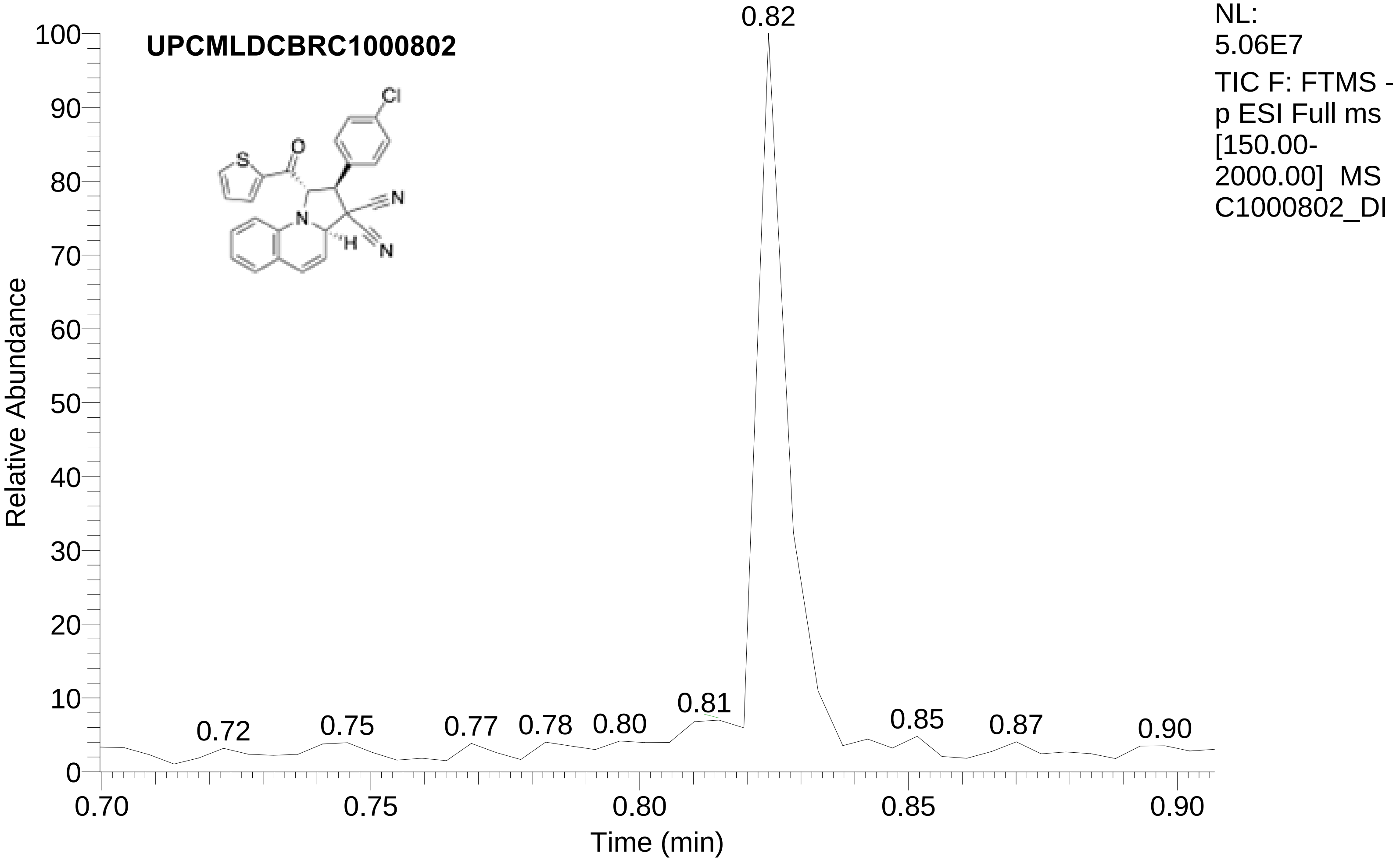

C1000802_DI #177-180 RT: 0.82-0.83 AV: 4 NL: 5.03E5
T: FTMS - p ESI Full ms [150.00-2000.00]

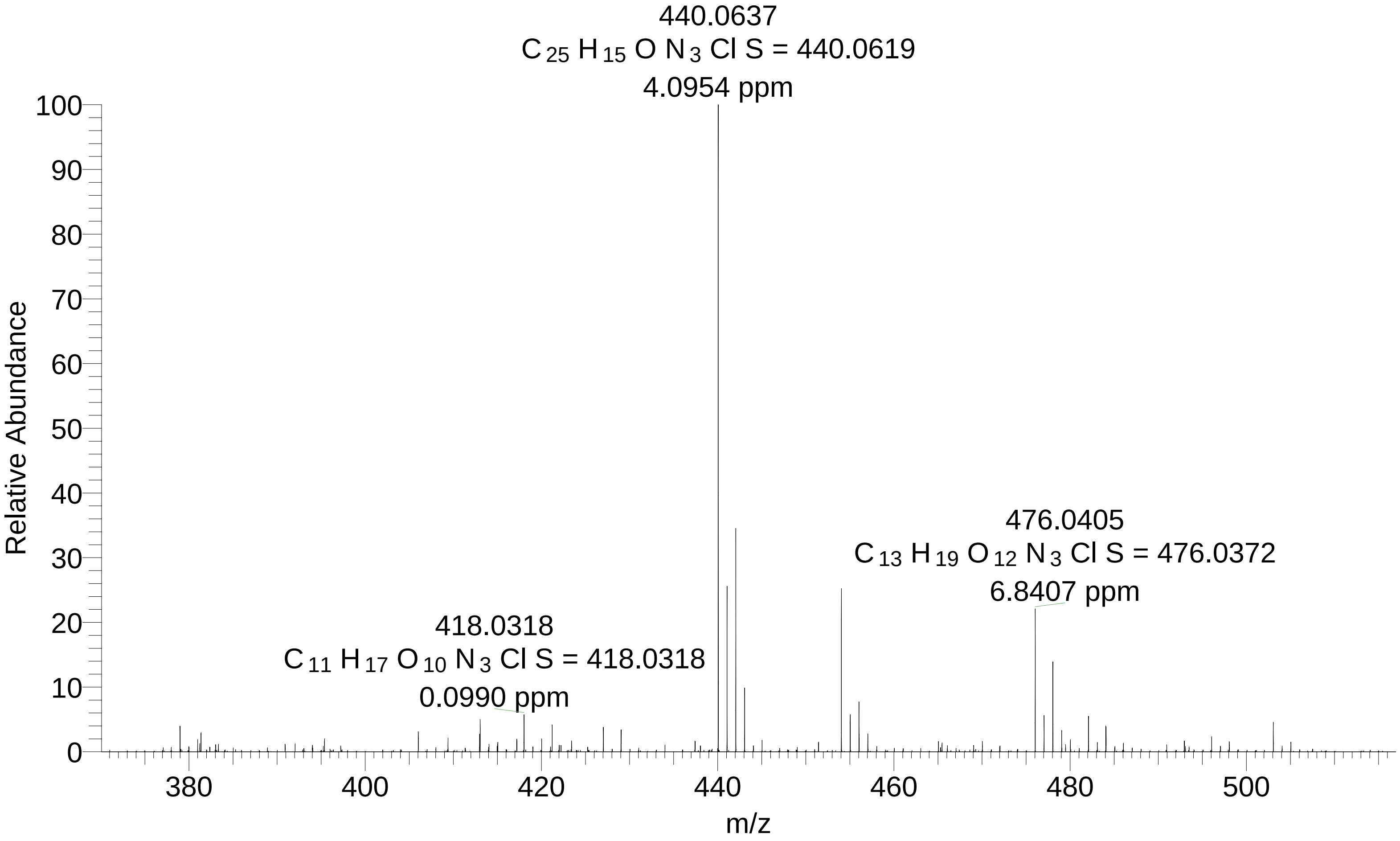

D:\data\Pete\C1001299_1 6/4/2018 11:30:56 AM **UPCMLDCBRC1001299**
C:\Xcalibur\methods\walkup\10min_0395A05M_pn.meth
RT: 0.00 - 9.98

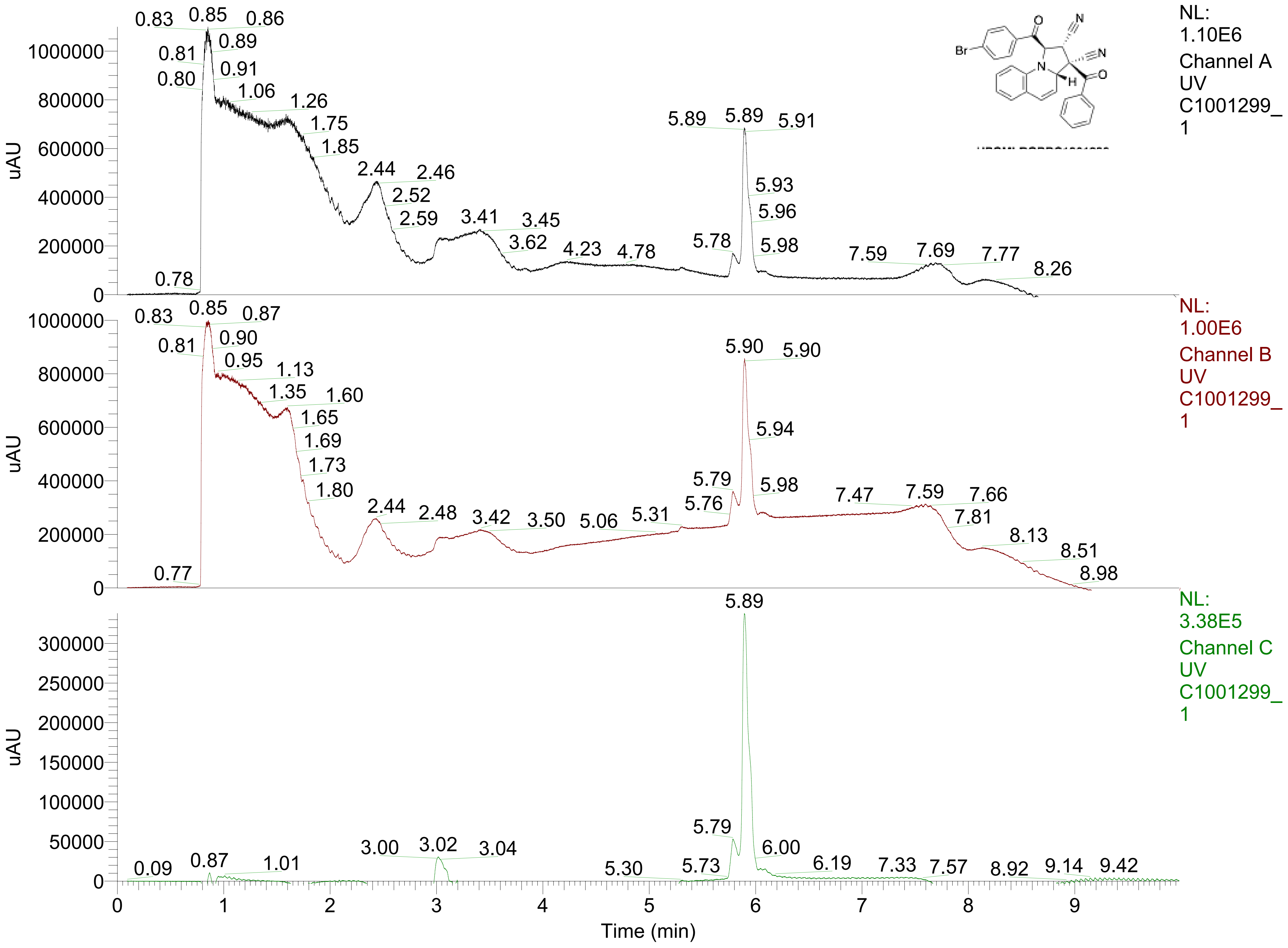

RT: 1.52 - 2.03

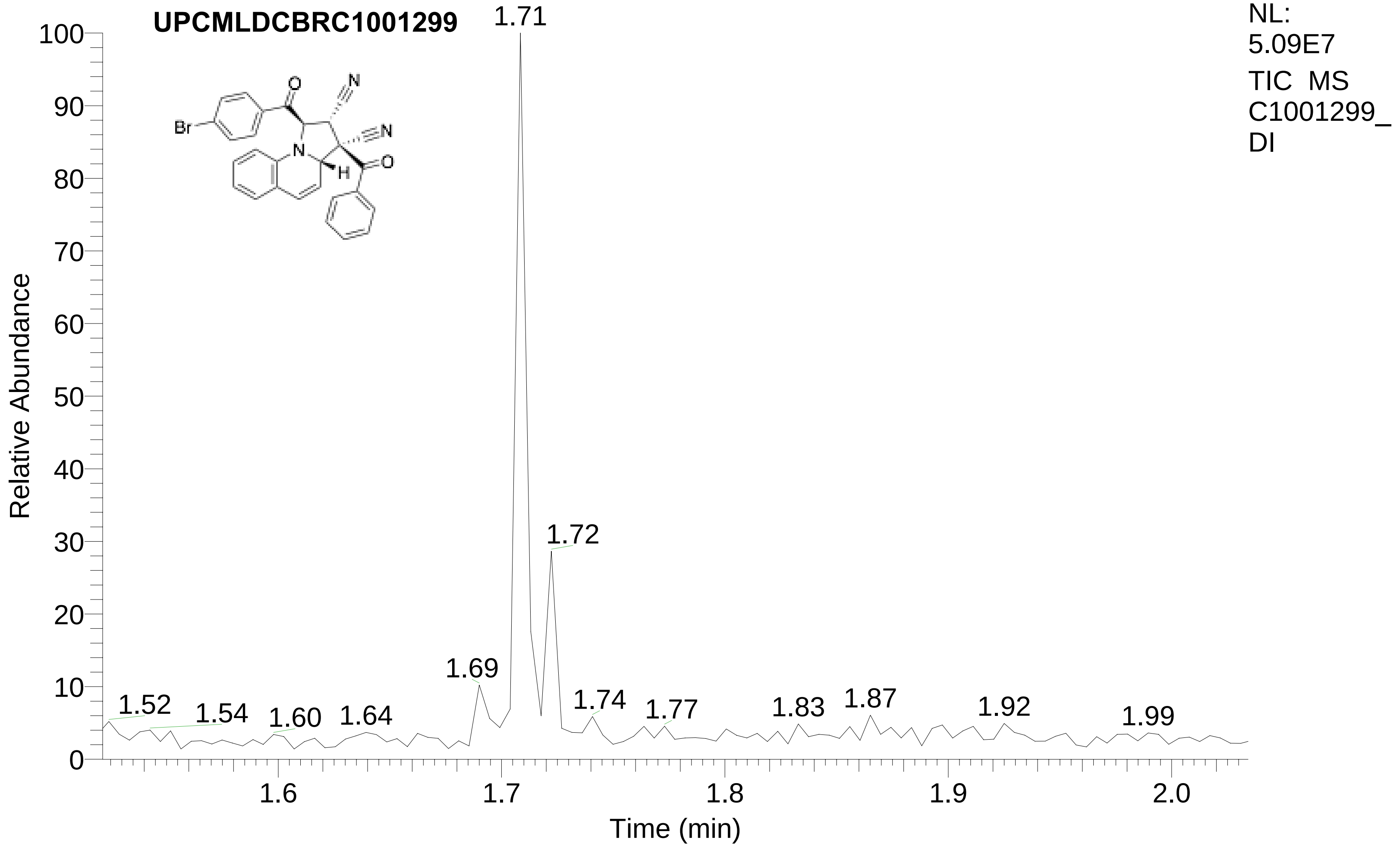

C1001299_DI #369 RT: 1.70 AV: 1 NL: 4.85E3
T: FTMS - p ESI Full ms [150.00-2000.00]

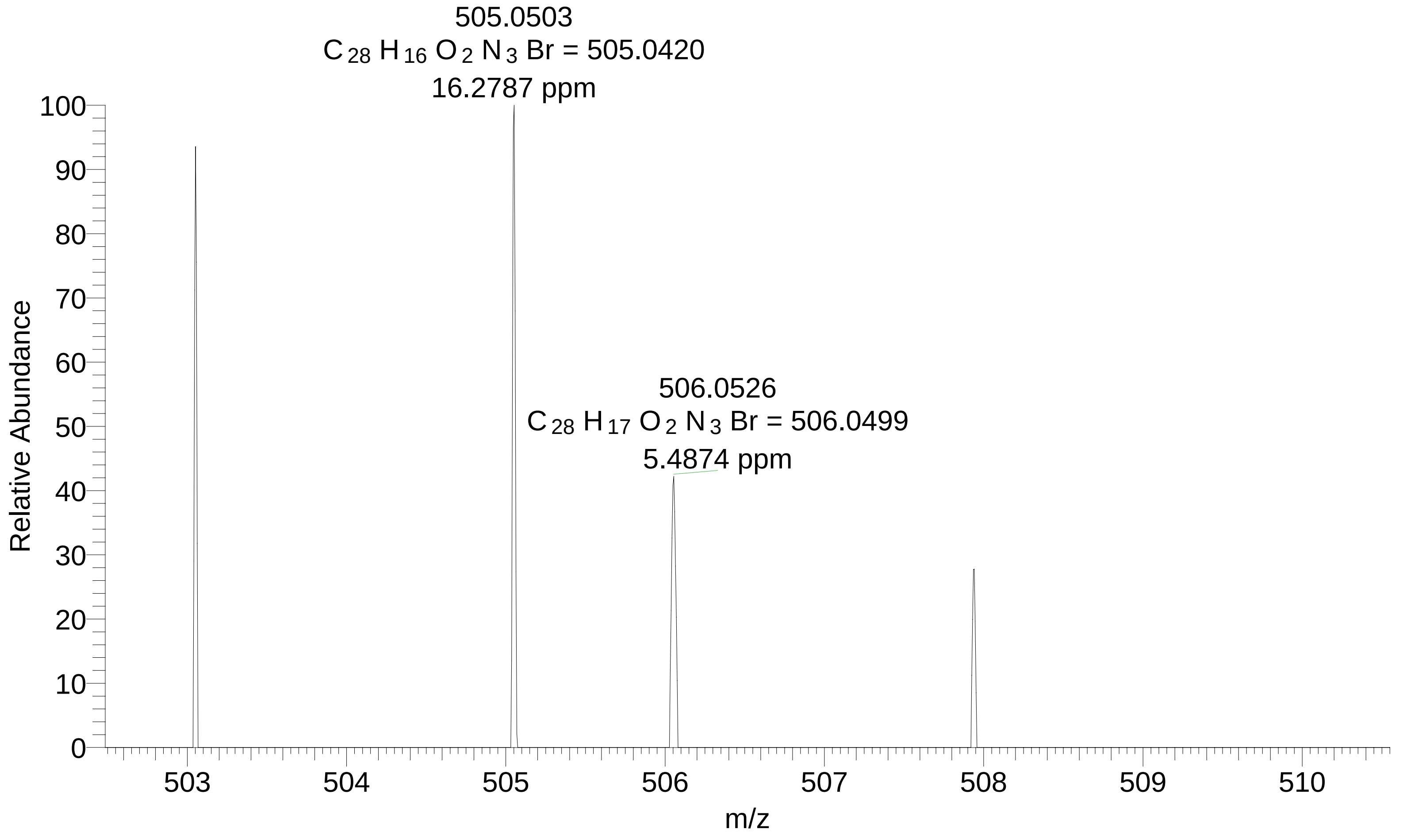

D:\data\Pete\C1002602_1 6/4/2018 11:41:57 AM **UPCMLDCBRC1002602**
C:\Xcalibur\methods\walkup\10min_0395A05M_pn.meth

RT: 0.00 - 9.98

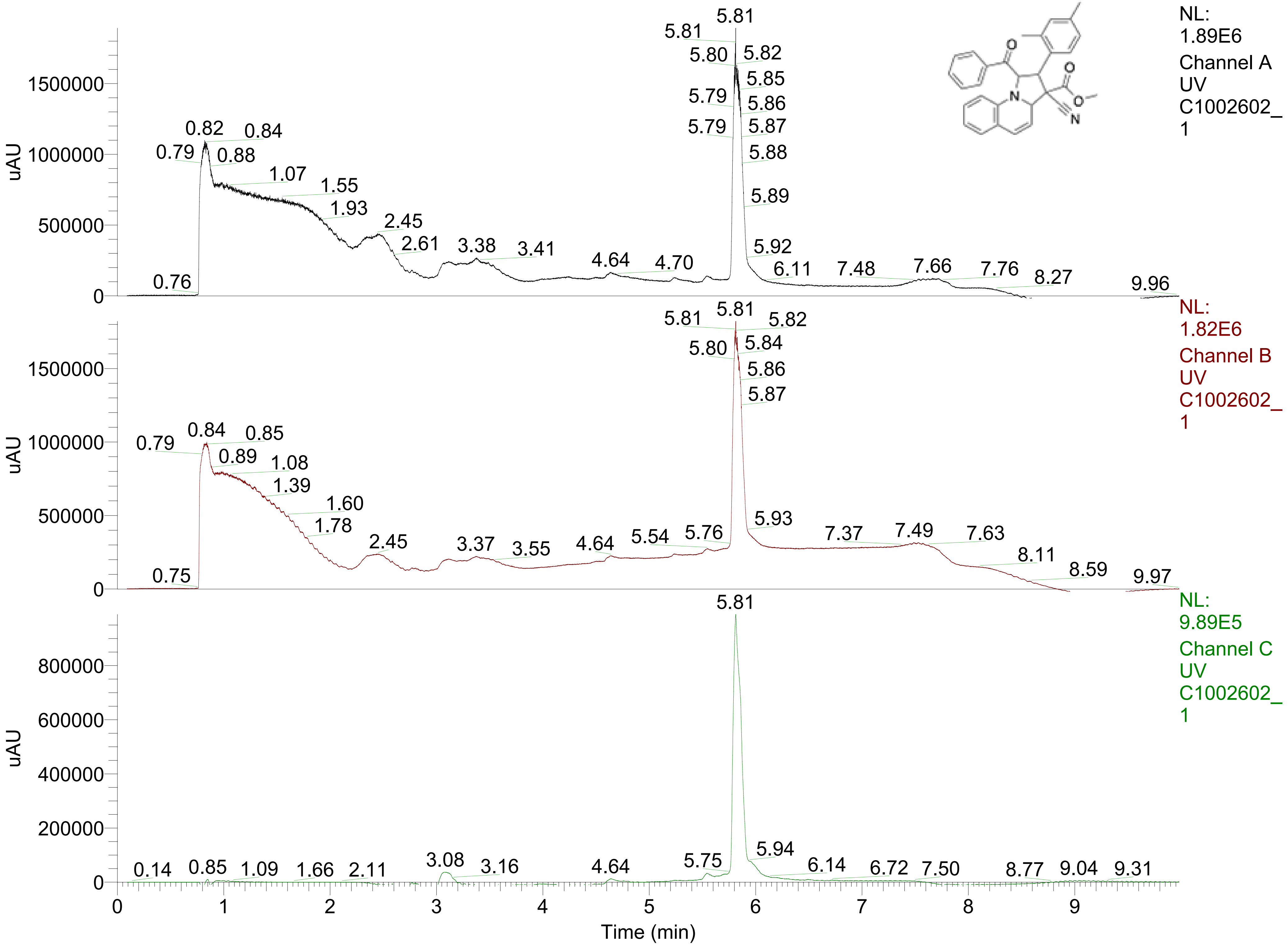

RT: 3.41 - 4.50

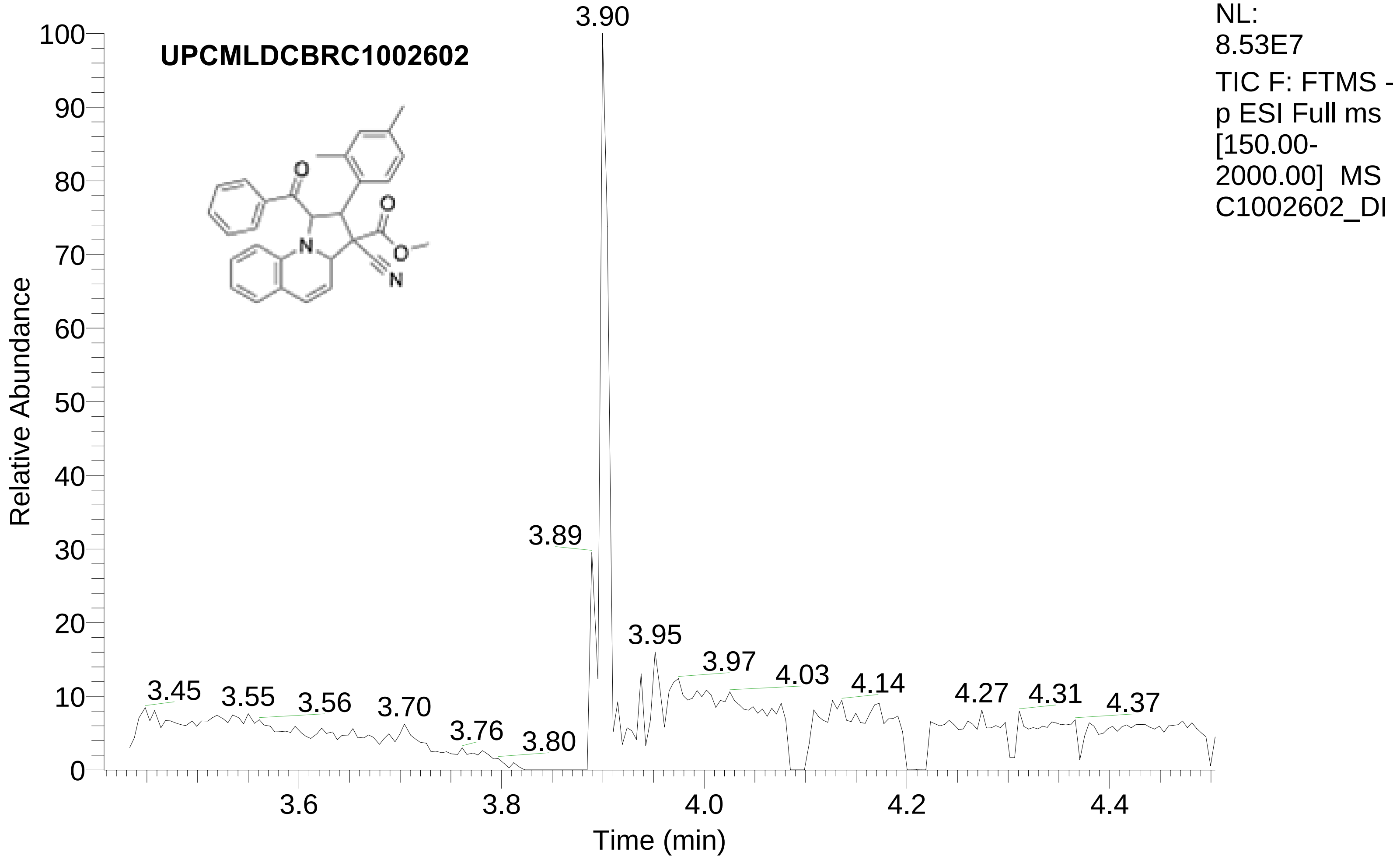

C1002602_DI #823-828 RT: 3.89-3.91 AV: 6 NL: 2.72E4
T: FTMS - p ESI Full ms [150.00-2000.00]

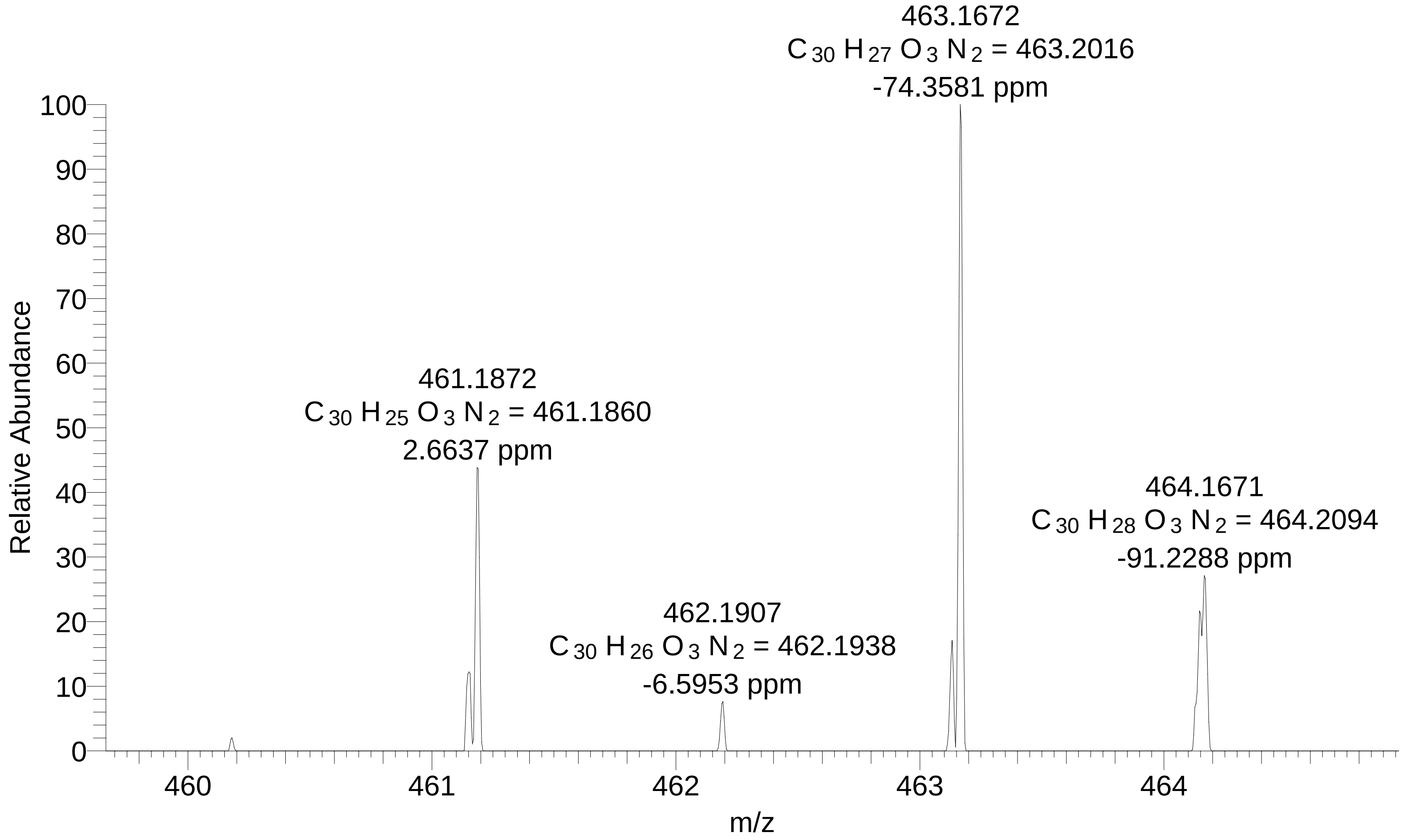

D:\data\Pete\C1004885_1
6/5/2018 9:14:27 AM
UPCMLDCBRC1004885
C:\Xcalibur\methods\walkup\10min_0395A05M_pn.meth
RT: 0.00 - 9.98

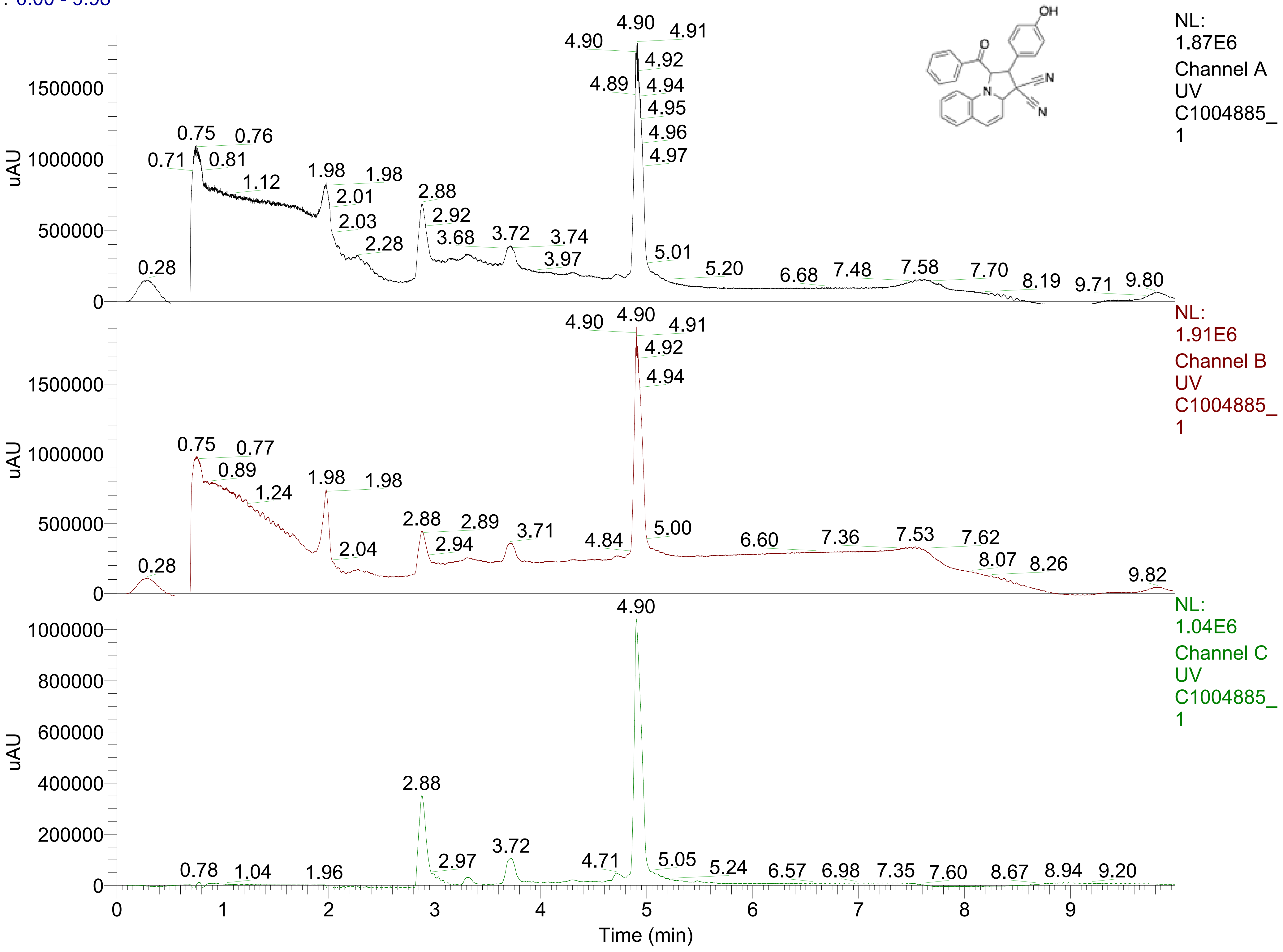

RT: 0.09 - 0.54

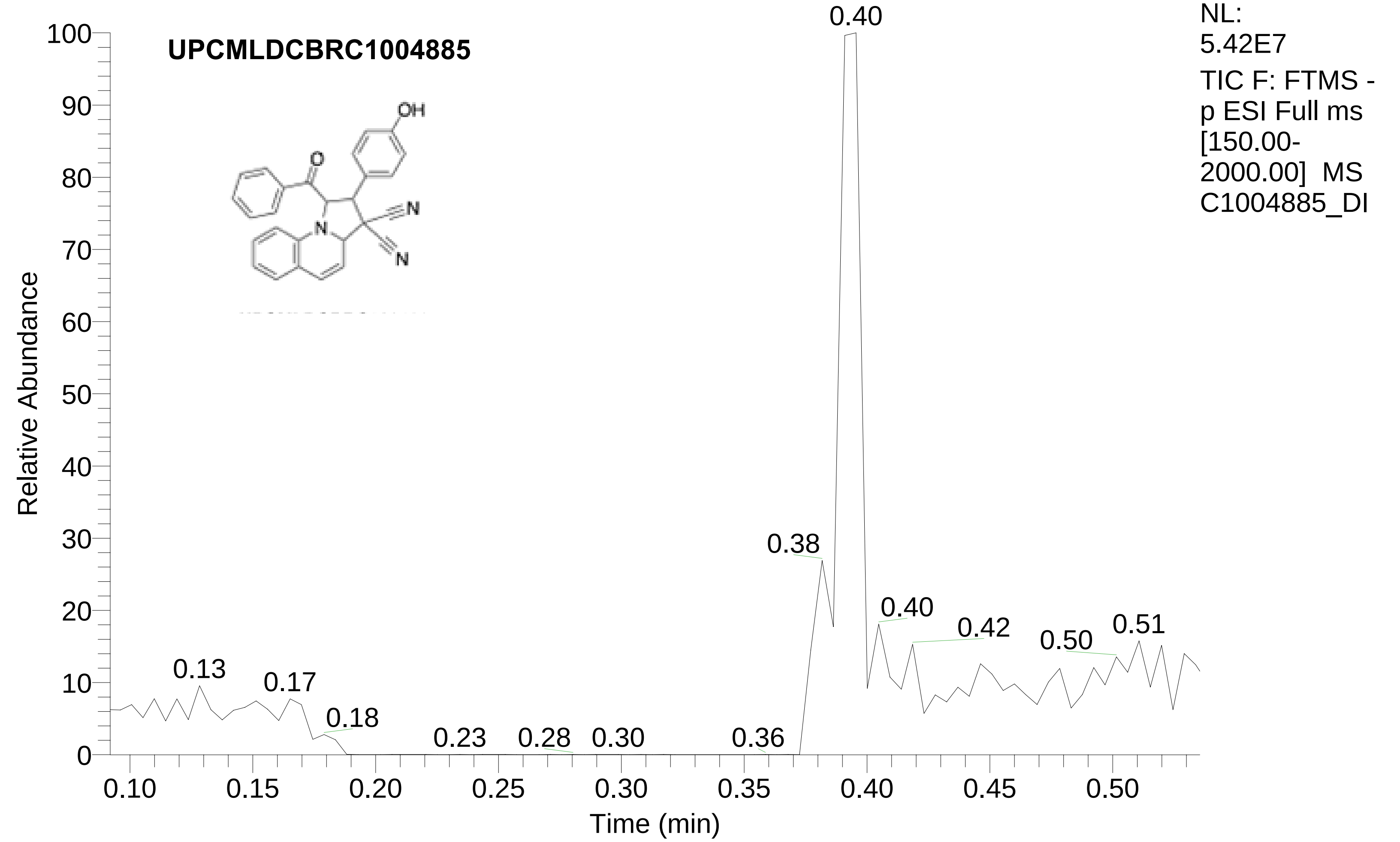

C1004885_DI #83-85 RT: 0.39-0.40 AV: 3 NL: 1.98E6
T: FTMS - p ESI Full ms [150.00-2000.00]

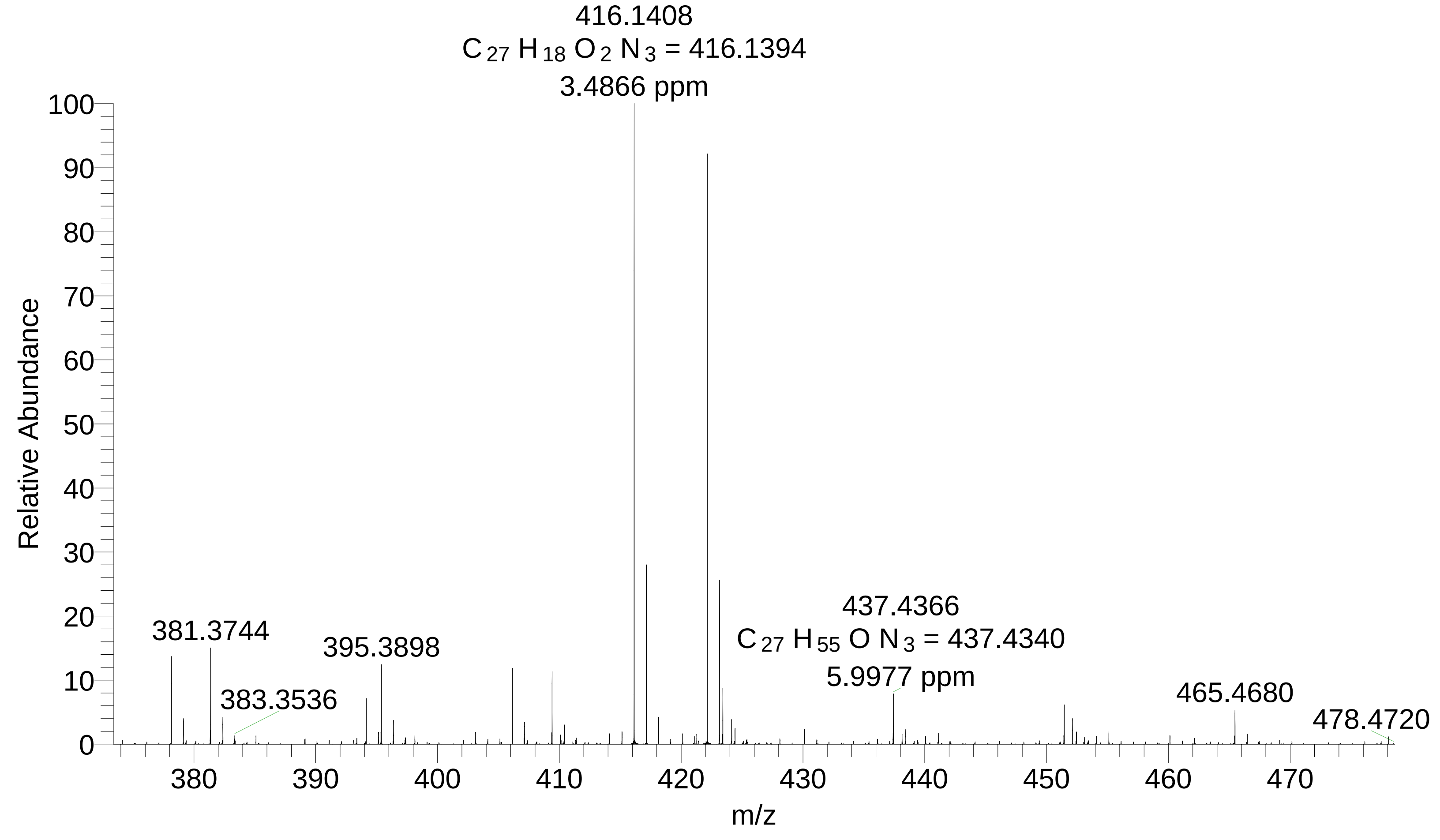

D:\data\Pete\C1033038_3 6/5/2018 10:36:39 AM **UPCMLDCBRC1033038**

C:\Xcalibur\methods\walkup\10min_0395A05M_pn.meth

RT: 0.00 - 9.98

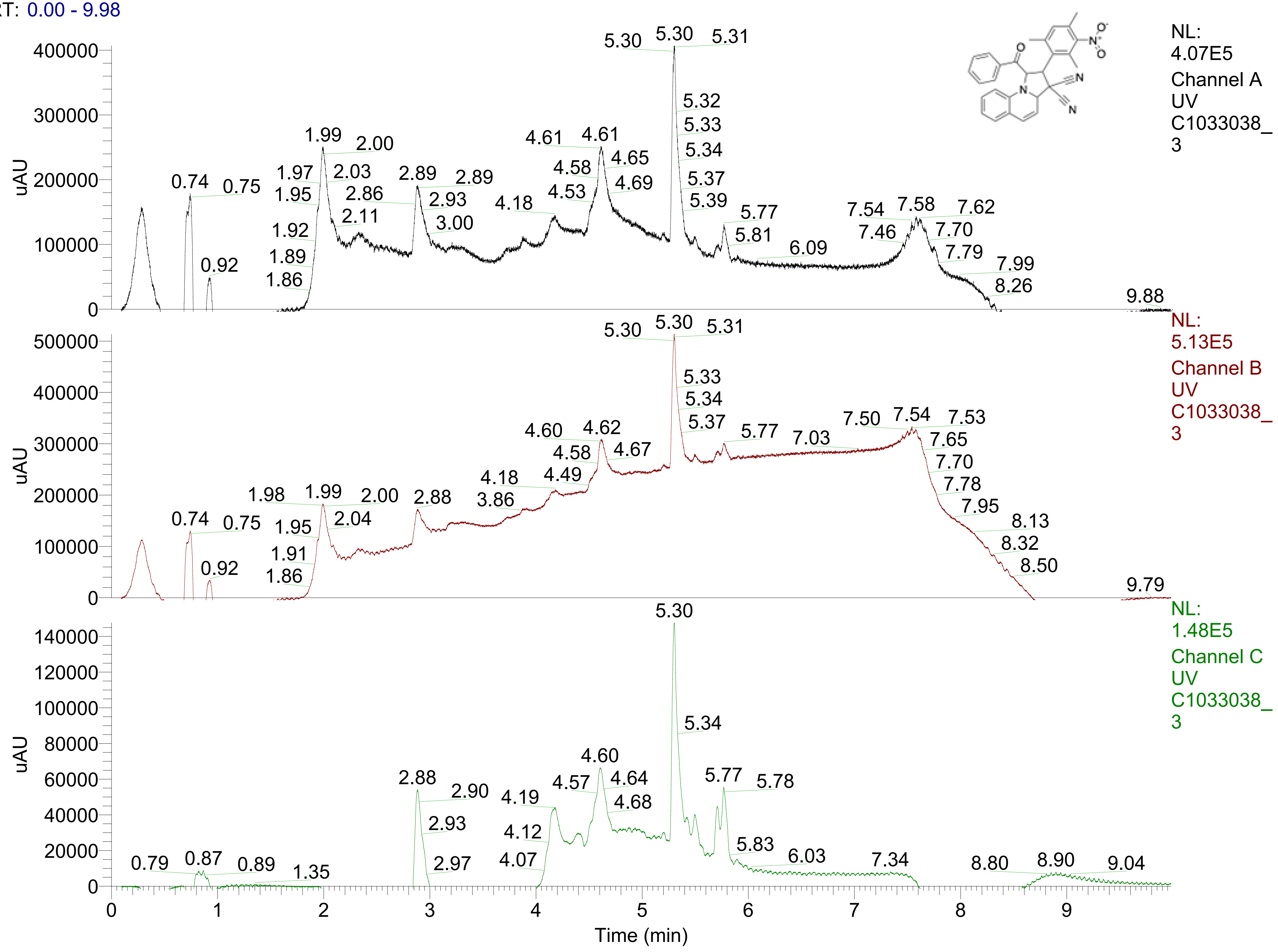

RT: 0.69 - 1.50

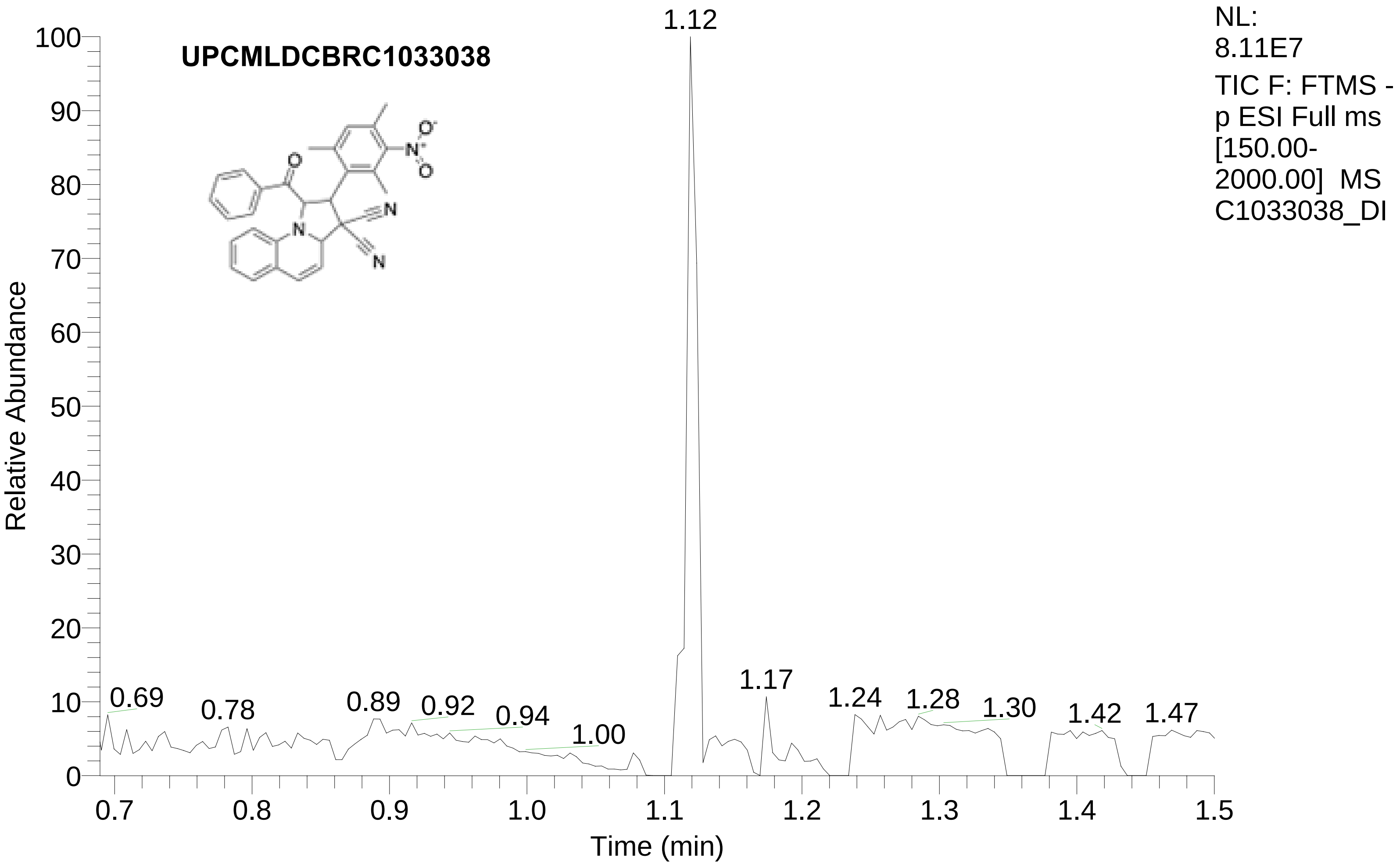

C1033038_DI #240-244 RT: 1.11-1.13 AV: 5 NL: 2.32E5
T: FTMS - p ESI Full ms [150.00-2000.00]

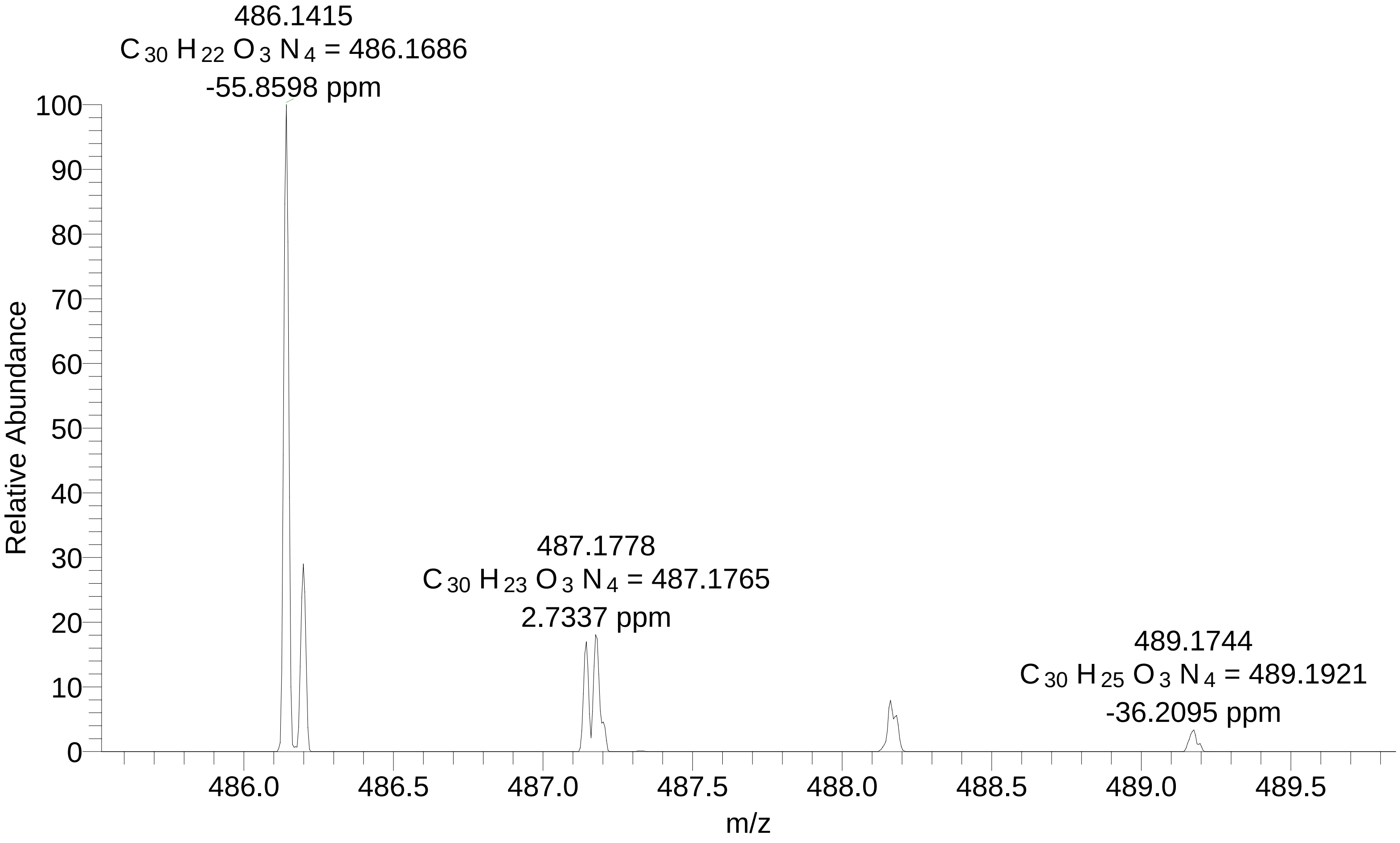

D:\data\Pete\C1033229_1
C:\Xcalibur\methods\walkup\10min_0395A05M_pn.meth

6/5/2018 9:36:27 AM

UPCMLDCBRC1033229

RT: 0.00 - 9.98

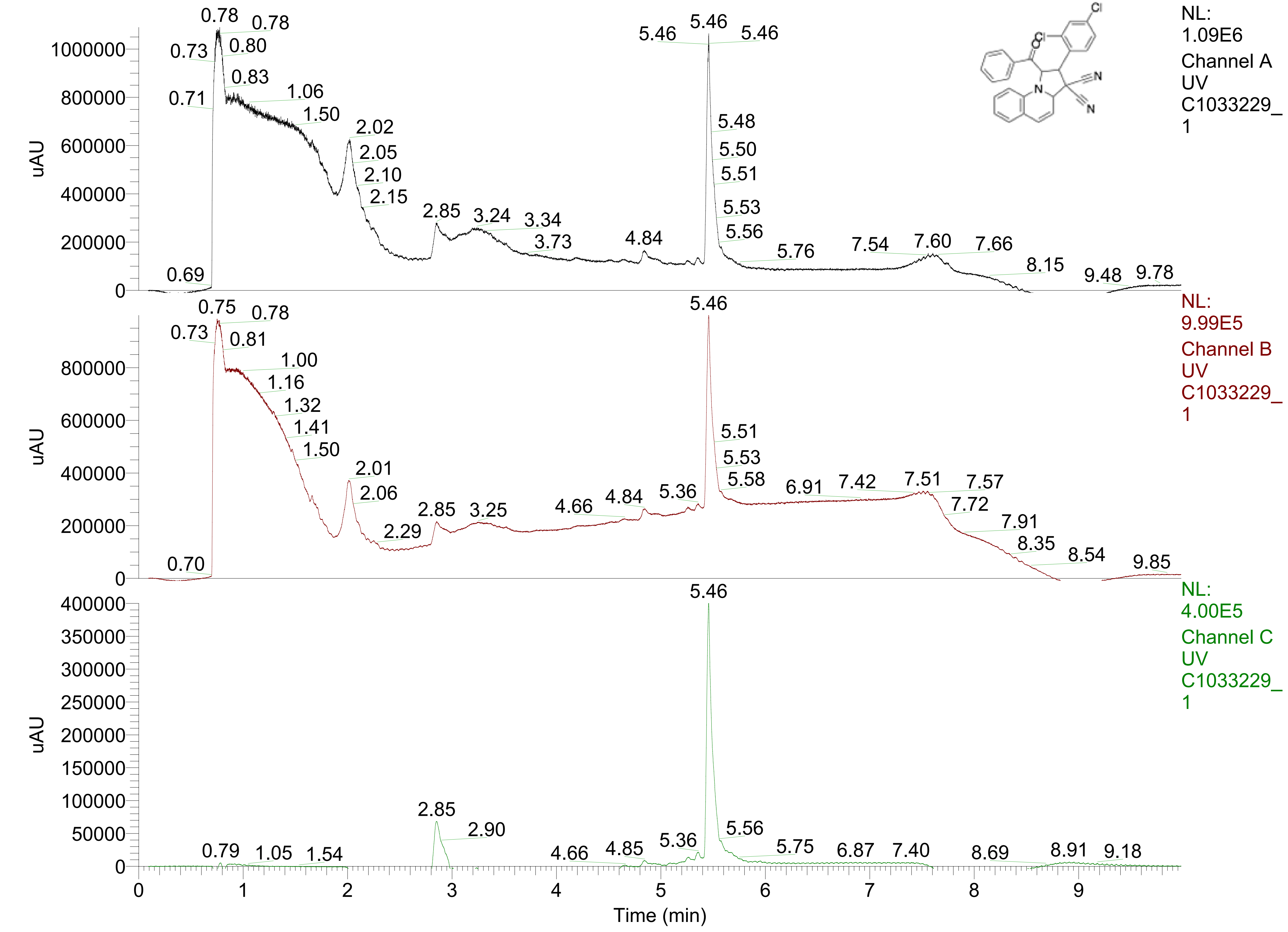

RT: 0.68 - 0.89

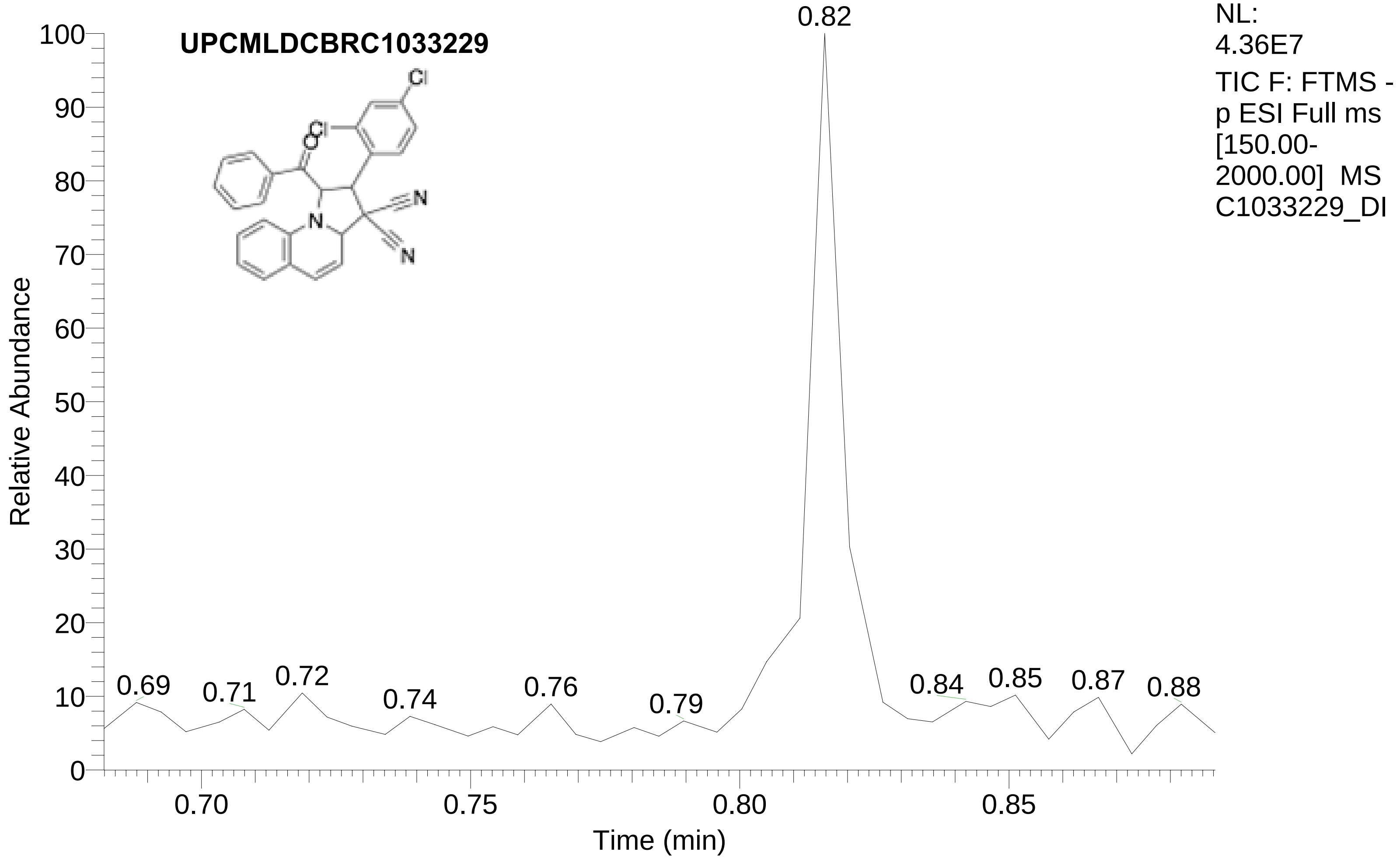

C1033229_DI #156-158 RT: 0.80-0.82 AV: 3 NL: 5.25E5
T: FTMS - p ESI Full ms [150.00-2000.00]

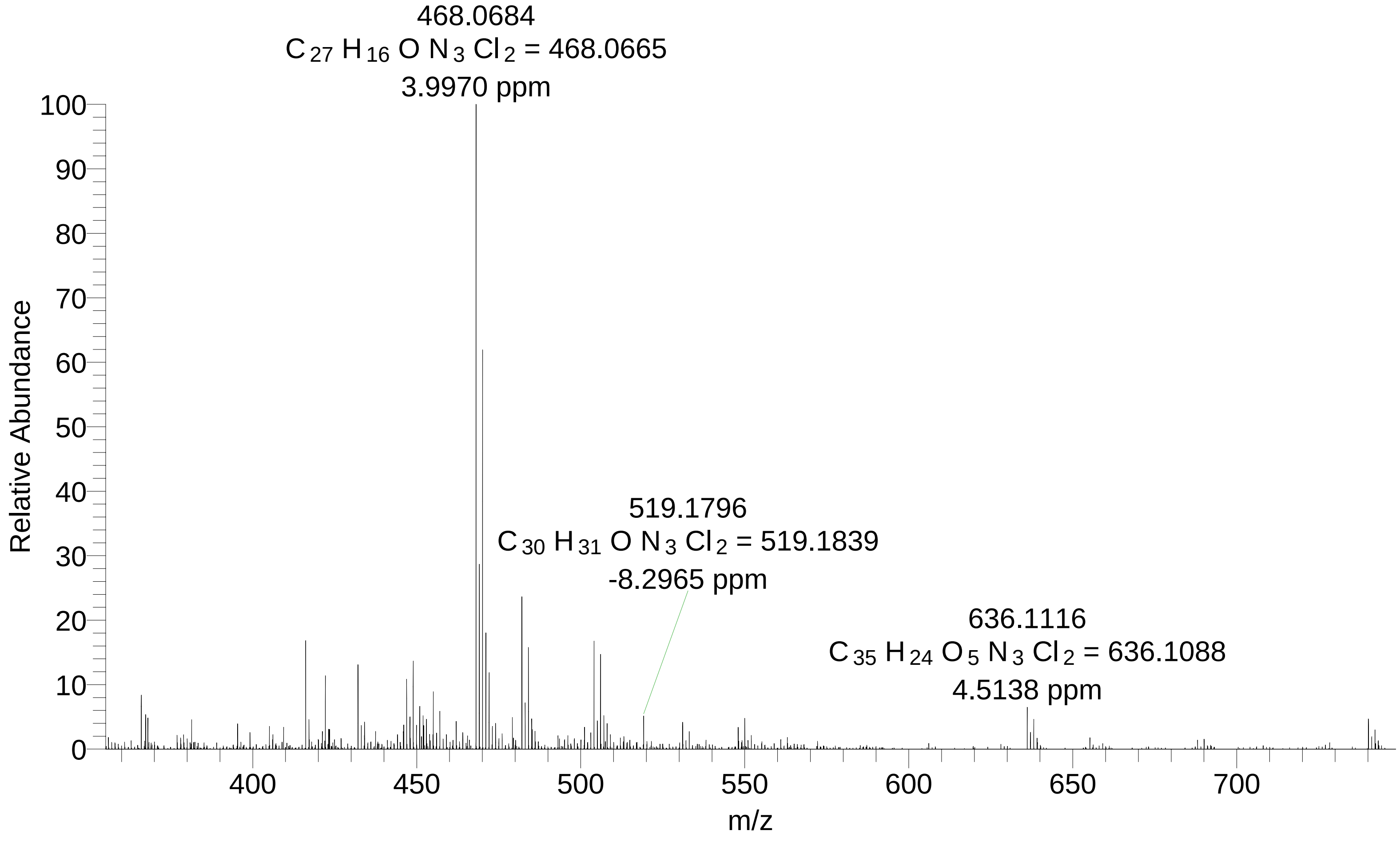

D:\data\Pete\C1038747_1 6/5/2018 9:47:27 AM **UPCMLDCBRC1038747**
C:\Xcalibur\methods\walkup\10min_0395A05M_pn.meth

RT: 0.00 - 9.98

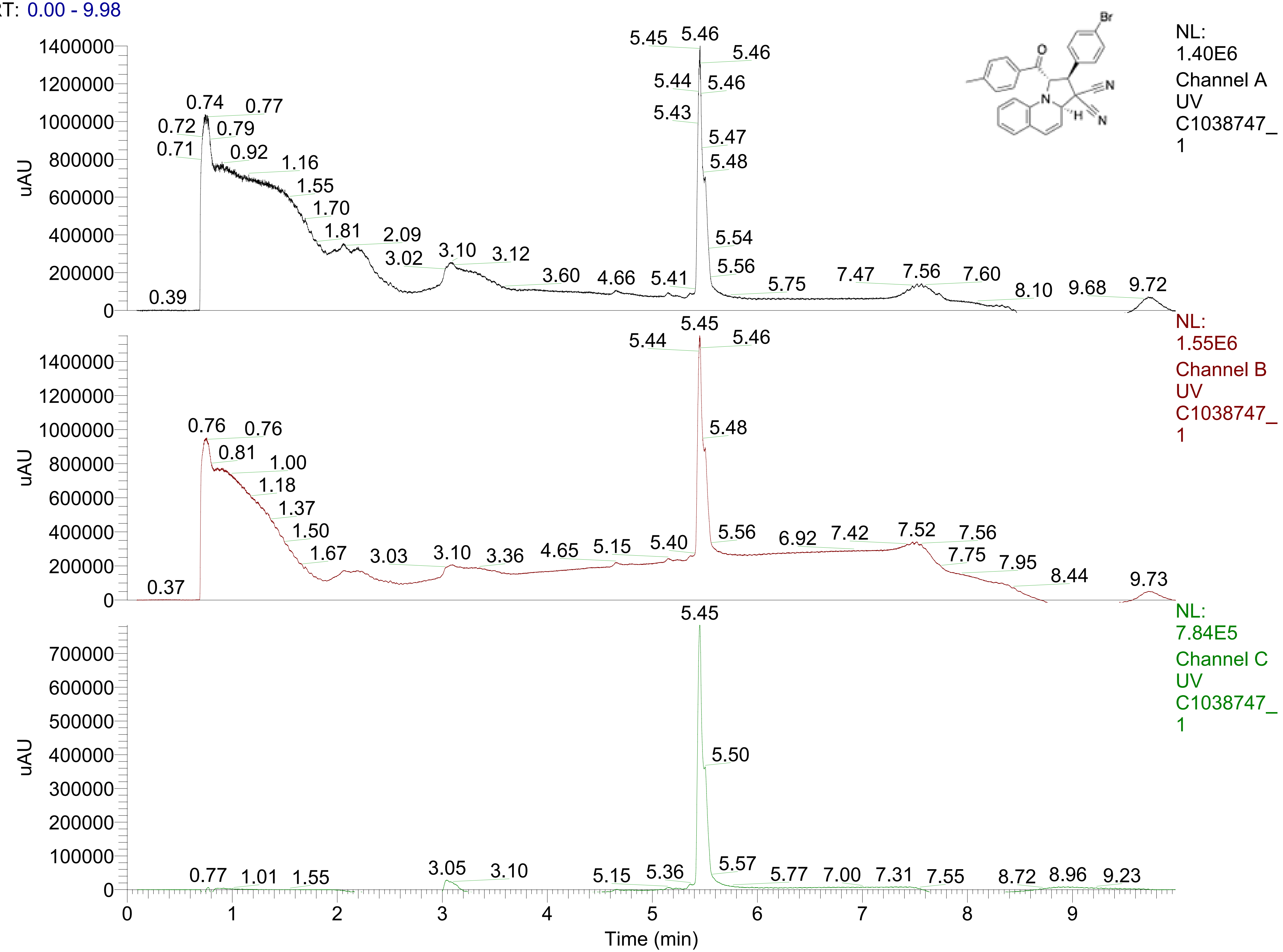

RT: 0.60 - 1.23

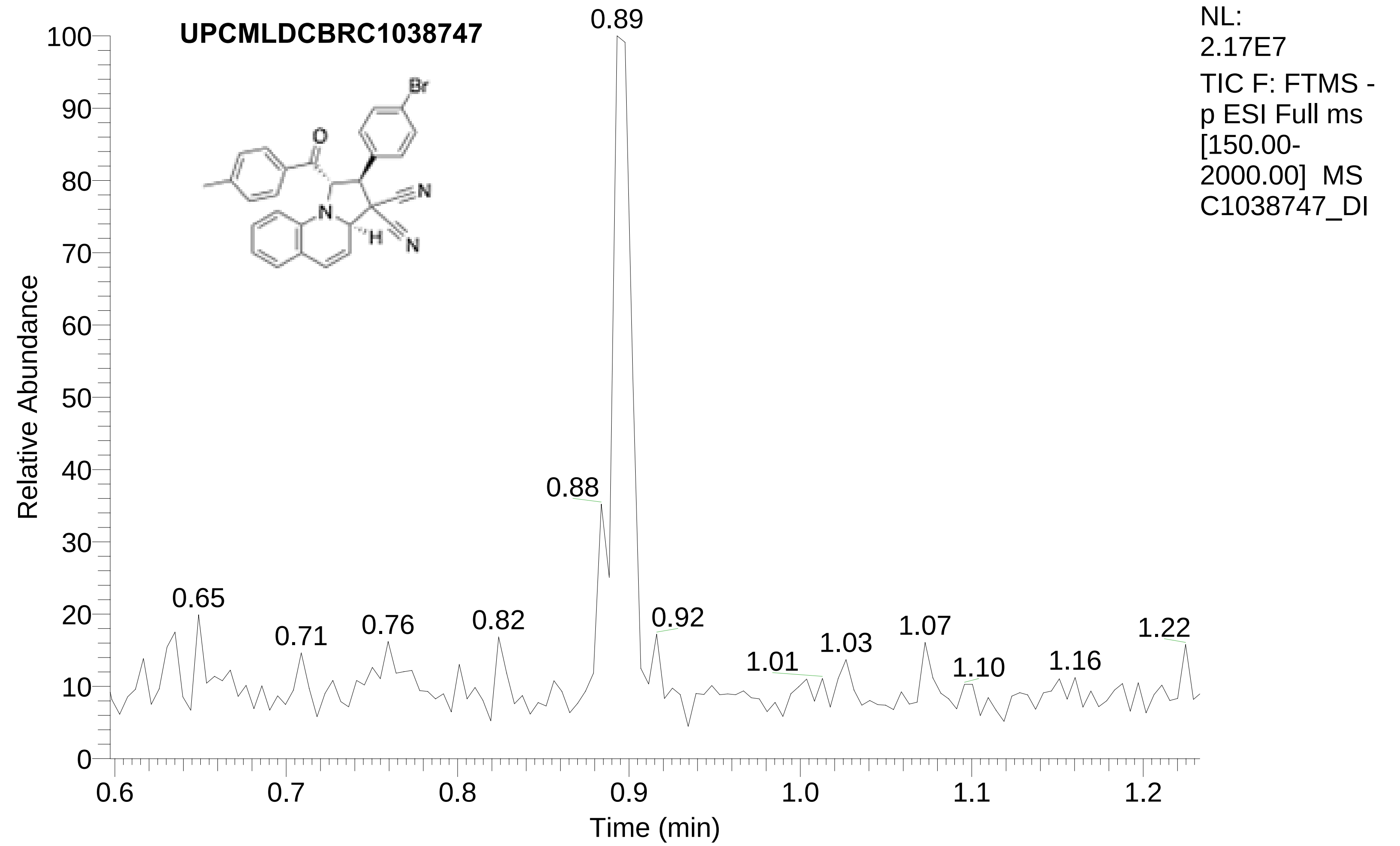

C1038747_DI #190-196 RT: 0.88-0.91 AV: 7 NL: 4.15E5
T: FTMS - p ESI Full ms [150.00-2000.00]

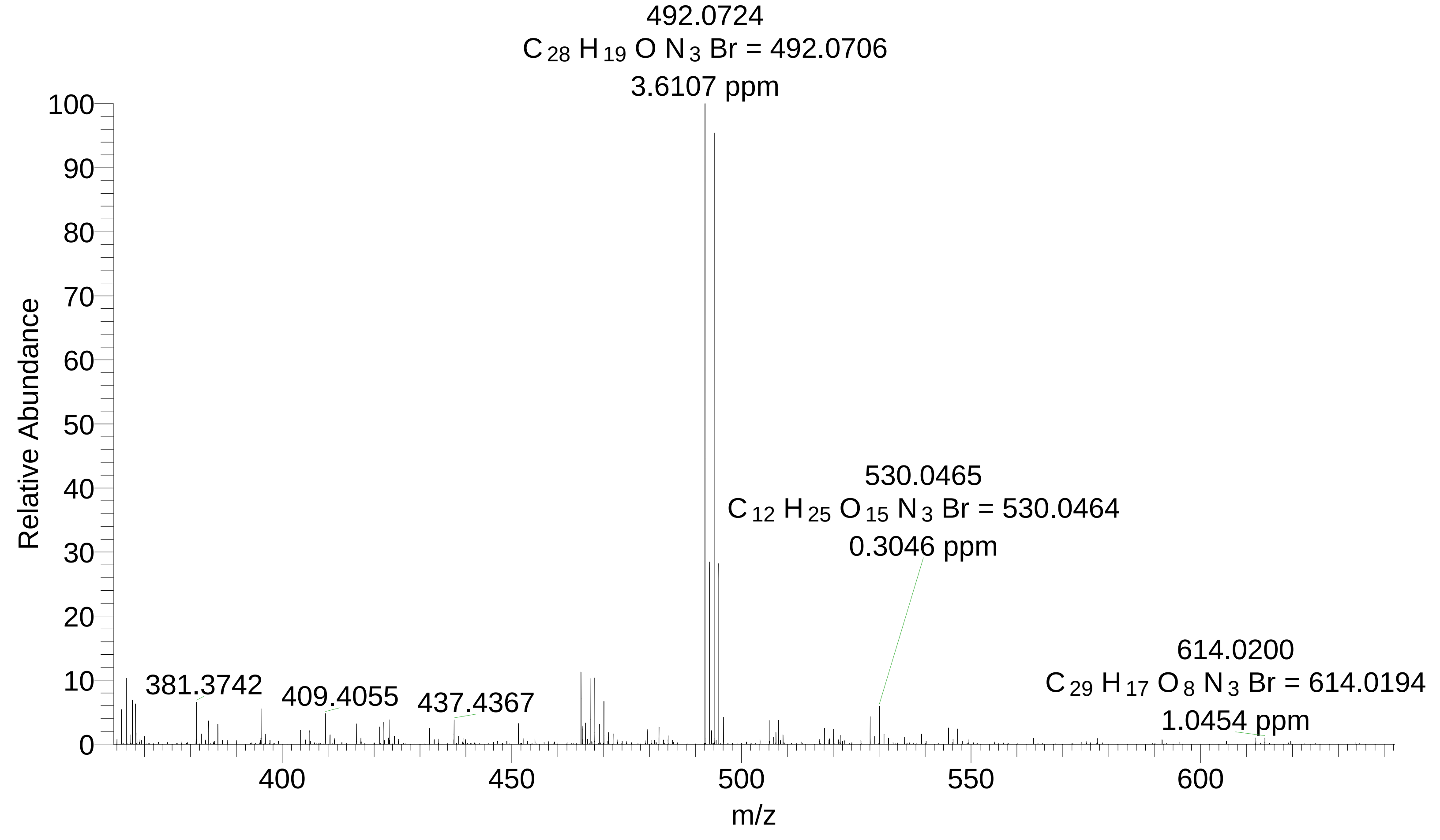

Supplement: Supplementary file 1 [file molecules-23-01691-s001.zip › molecules-327113 Supplementary Figures & Data/Supplementry data 4 timtecR.pdf]
