# Supplementary material for: A High-Content Screen Reveals New Small-Molecule Enhancers of Ras/Mapk Signaling as Probes for Zebrafish Heart Development
Source: Molecules. 2018 Jul 11;23(7):1691. doi: 10.3390/molecules23071691 (PMC6099644; doi:10.3390/molecules23071691)
Supplement: Supplementary file 1 [file molecules-23-01691-s001.zip › molecules-327113 Supplementary Figures & Data/Supplementary Data 3 timtecR.pdf]

D:\data\Pete\ST06994_neat_1_180222110548 2/22/2018 11:05:48 AM
C:\Xcalibur\methods\walkup\10min_0395A05M_pn.meth

RT: 0.00 - 9.98

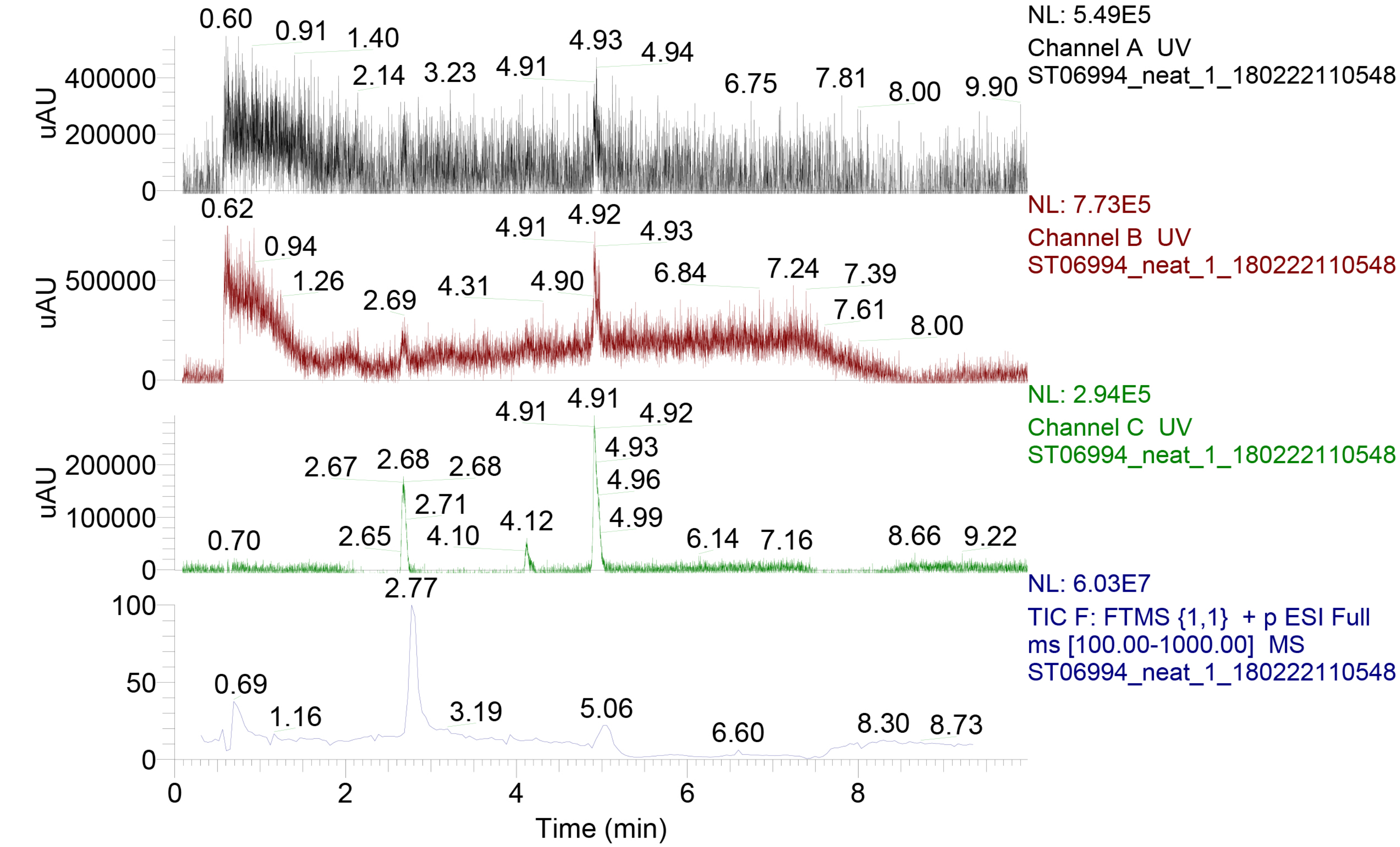

ST06994_neat_1_180222110548 #217-221 RT: 4.97-5.06 AV: 3 NL: 2.84E6
T: FTMS {1,1} + p ESI Full lock ms [100.00-1000.00]

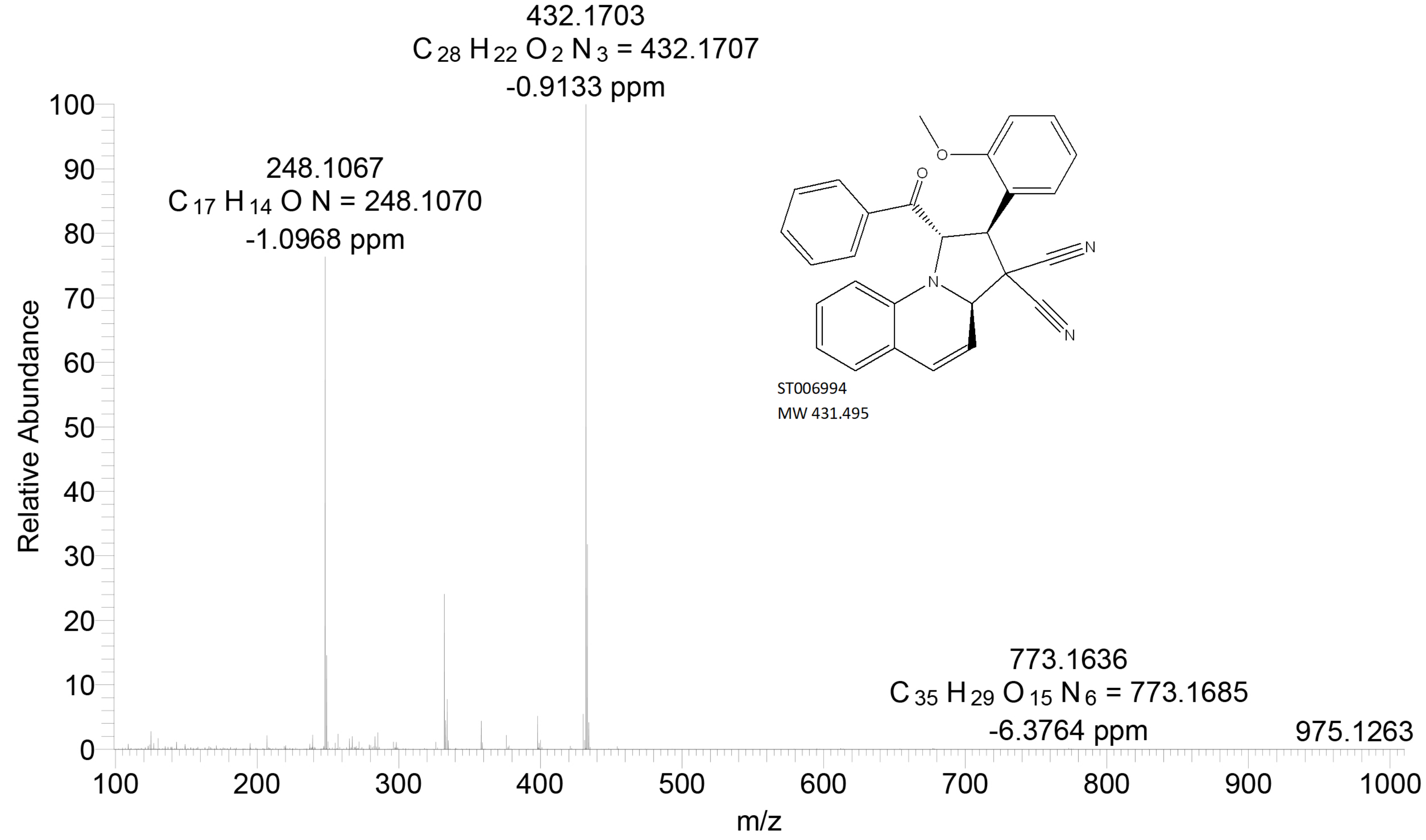

ST006994
MW 431.495

D:\data\Taber\ST020101_3 3/28/2018 7:35:08 AM
C:\Xcalibur\methods\walkup\10min_0395A05M_pn.meth

RT: 0.00 - 9.98

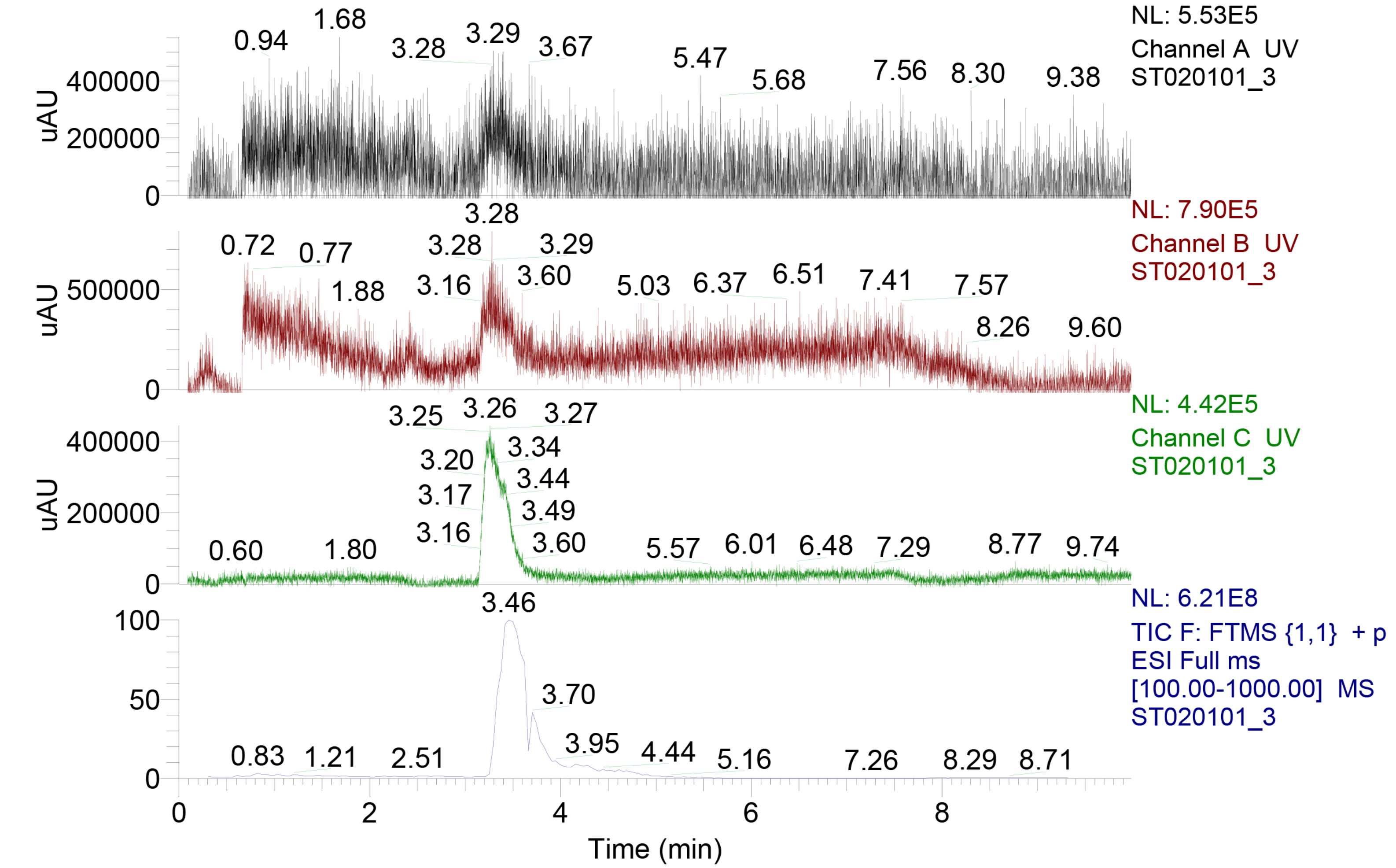

ST020101_3 #142-153 RT: 3.38-3.58 AV: 6 NL: 3.13E8
T: FTMS {1,1} + p ESI Full ms [100.00-1000.00]

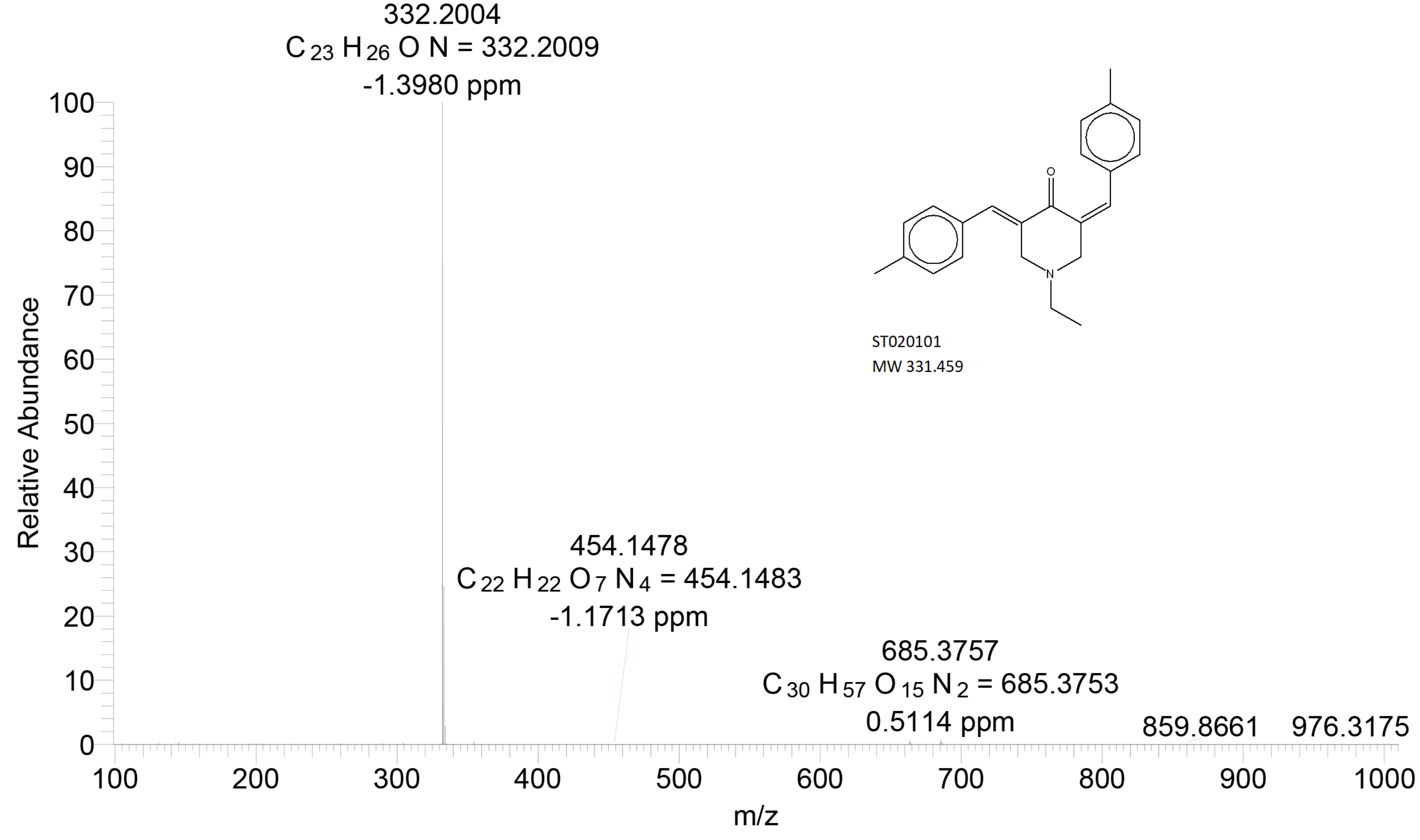

D:\data\Pete\Timtec_ST0011282_2 2/8/2018 10:28:53 AM
C:\Xcalibur\methods\walkup\10min_0395A05M_pn.meth

RT: 0.00 - 9.98

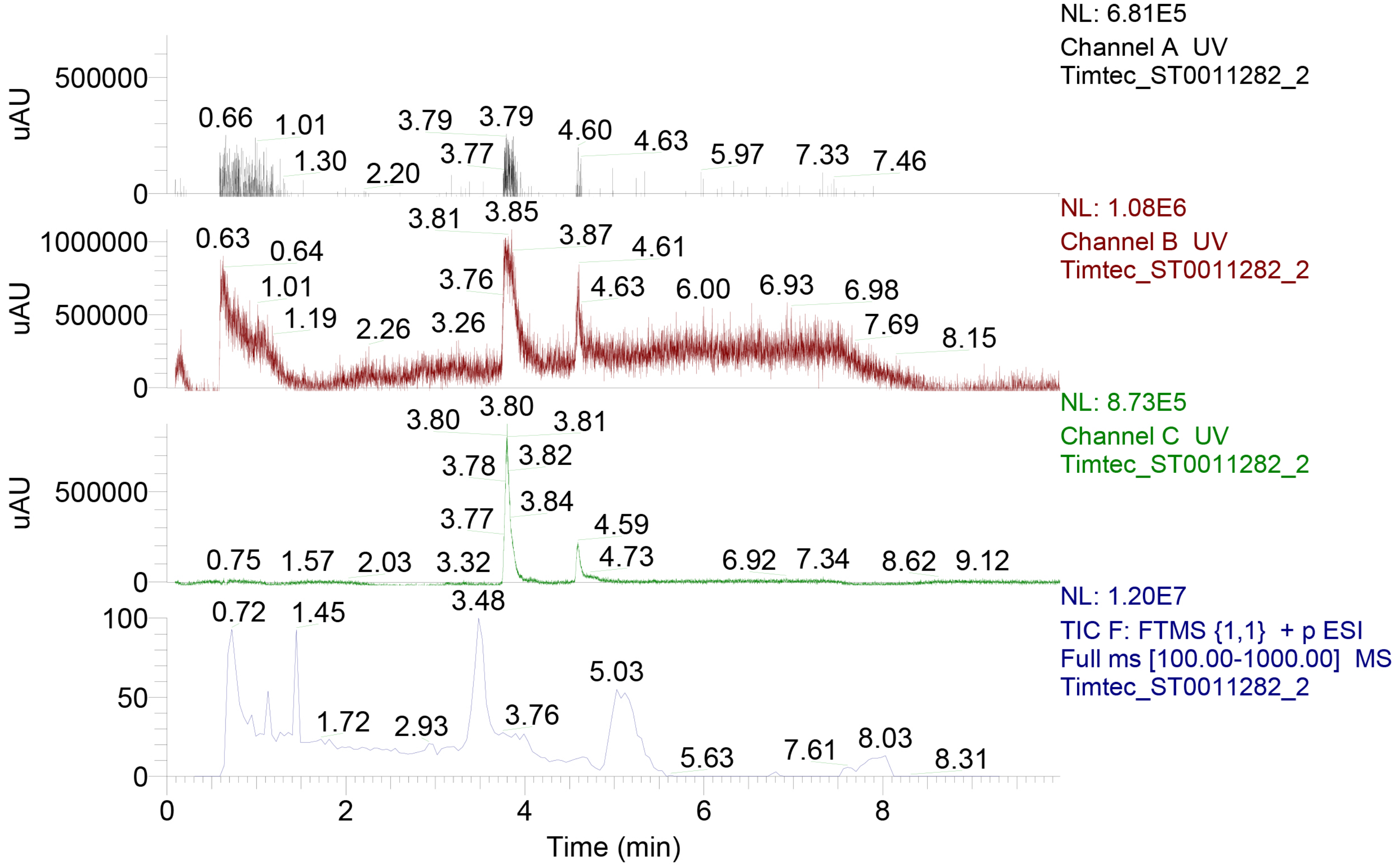

NL: 2.90E5
Timtec_ST0011282_2#149-159 RT: 3.71-3.94 AV: 6
T: FTMS {1,1} + p ESI Full lock ms [100.00-1000.00]

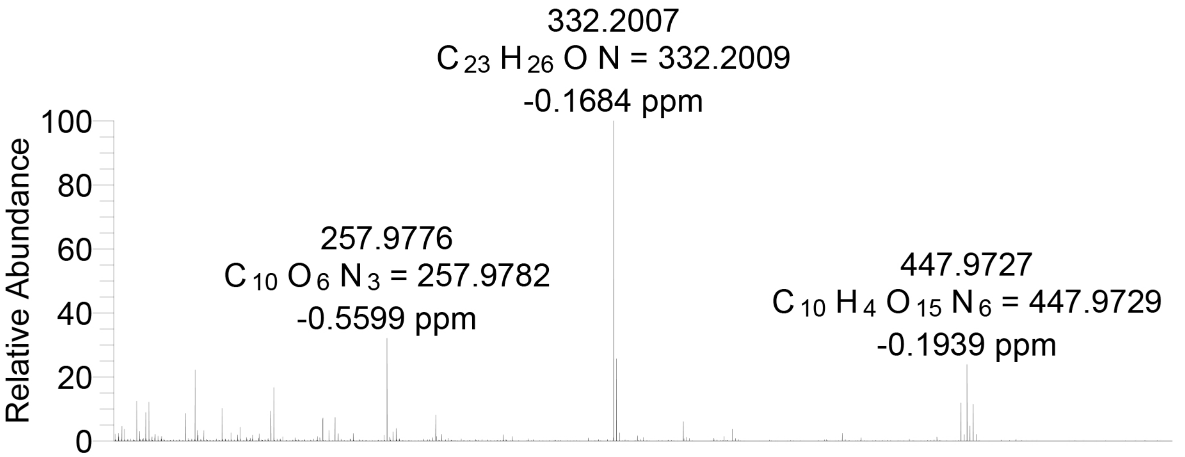

NL: 1.77E5
Timtec_ST0011282_2#202-208 RT: 4.98-5.07 AV: 3
T: FTMS {1,1} + p ESI Full lock ms [100.00-1000.00]

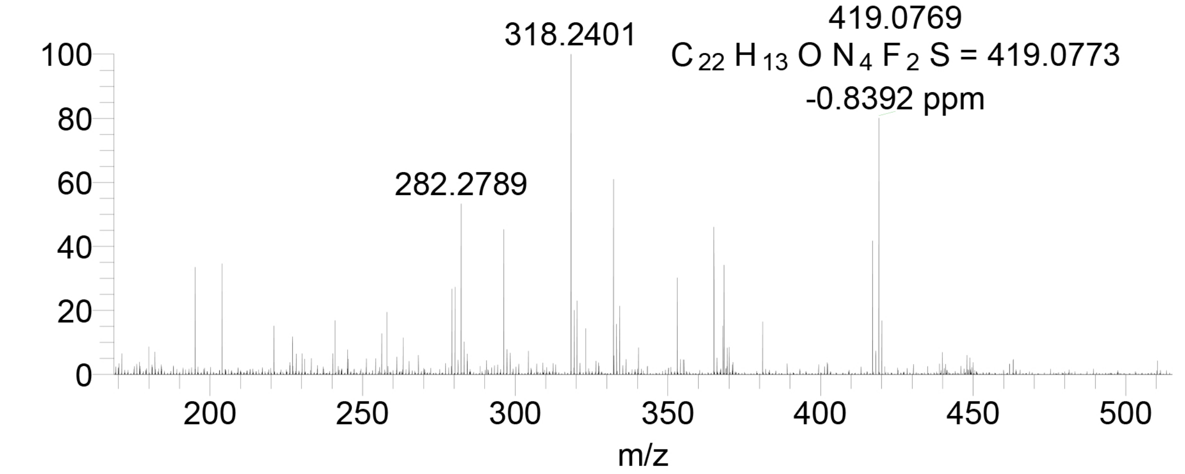

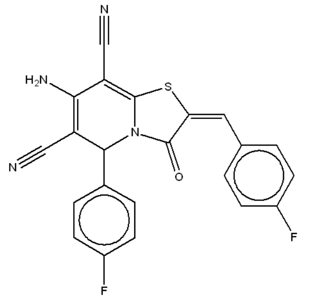

ST011282
MW 418.43
