# Supplementary figures and images for: A High-Content Screen Reveals New Small-Molecule Enhancers of Ras/Mapk Signaling as Probes for Zebrafish Heart Development
Source: Molecules. 2018 Jul 11;23(7):1691. doi: 10.3390/molecules23071691 (PMC6099644; doi:10.3390/molecules23071691)

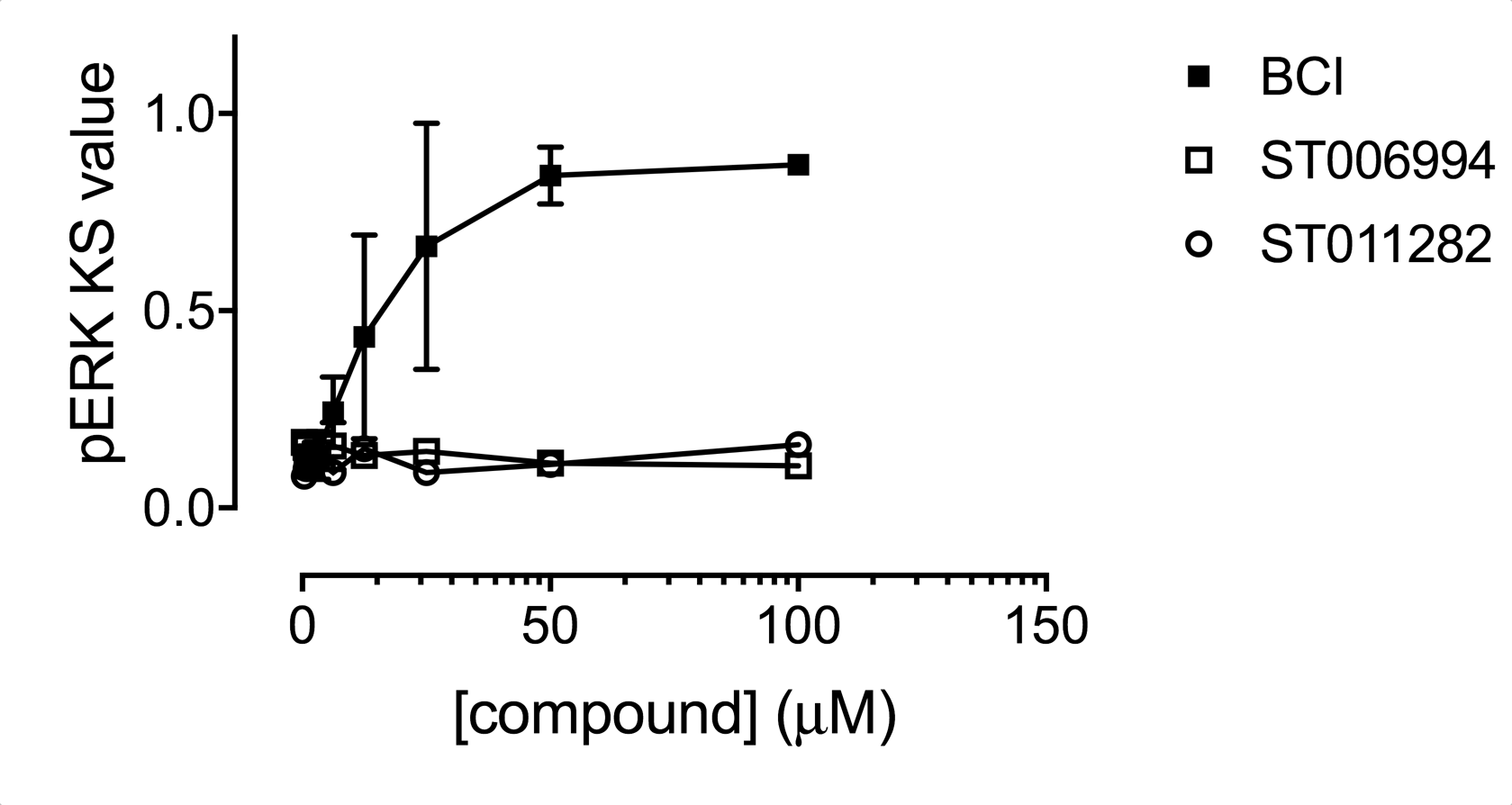

Supplement: Supplementary file 1 [file molecules-23-01691-s001.zip › molecules-327113 Supplementary Figures & Data/Supplementary Fig S4 timtecR.tiff]

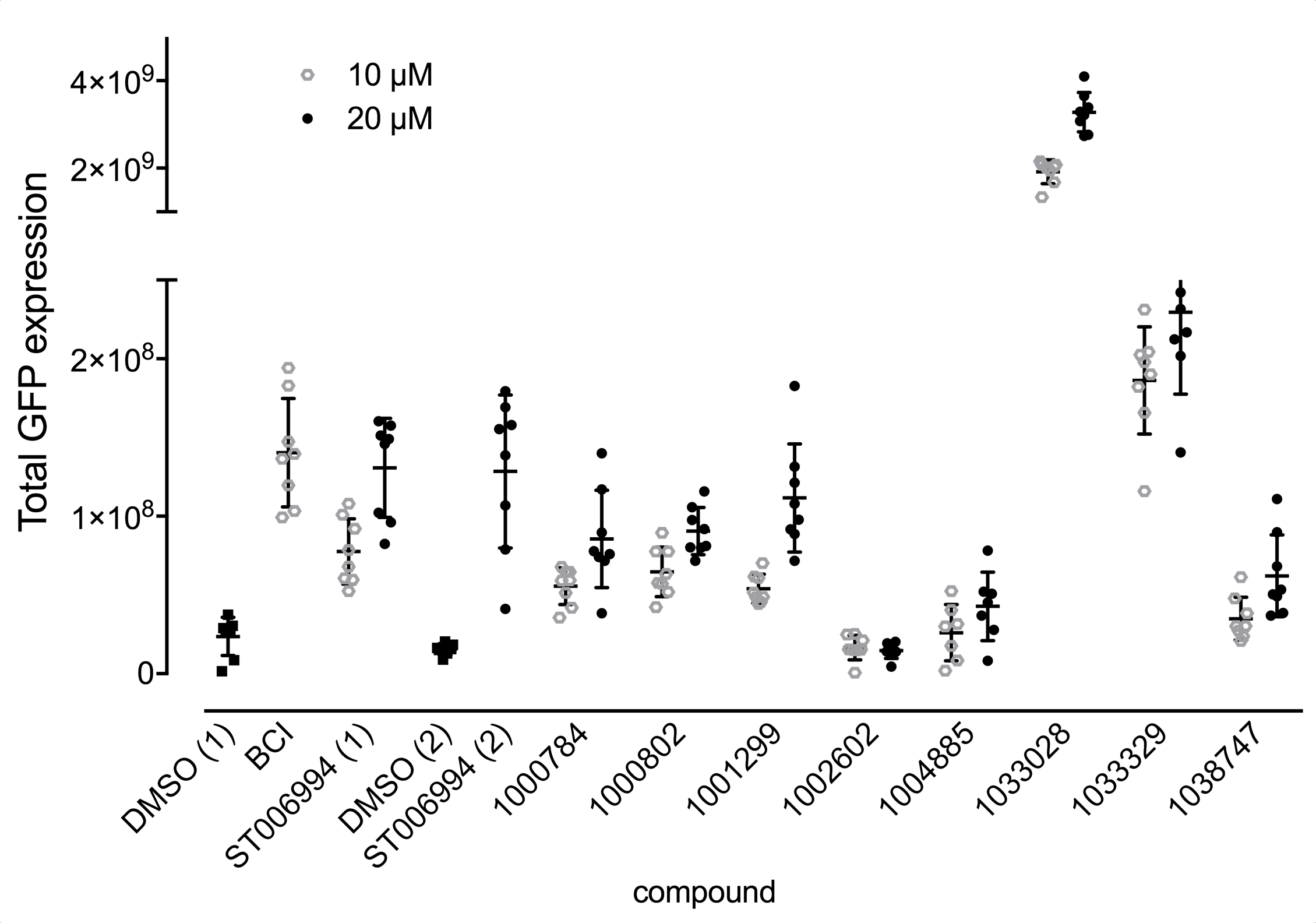

Supplement: Supplementary file 1 [file molecules-23-01691-s001.zip › molecules-327113 Supplementary Figures & Data/Supplementary Fig S3 timtecR.tiff]

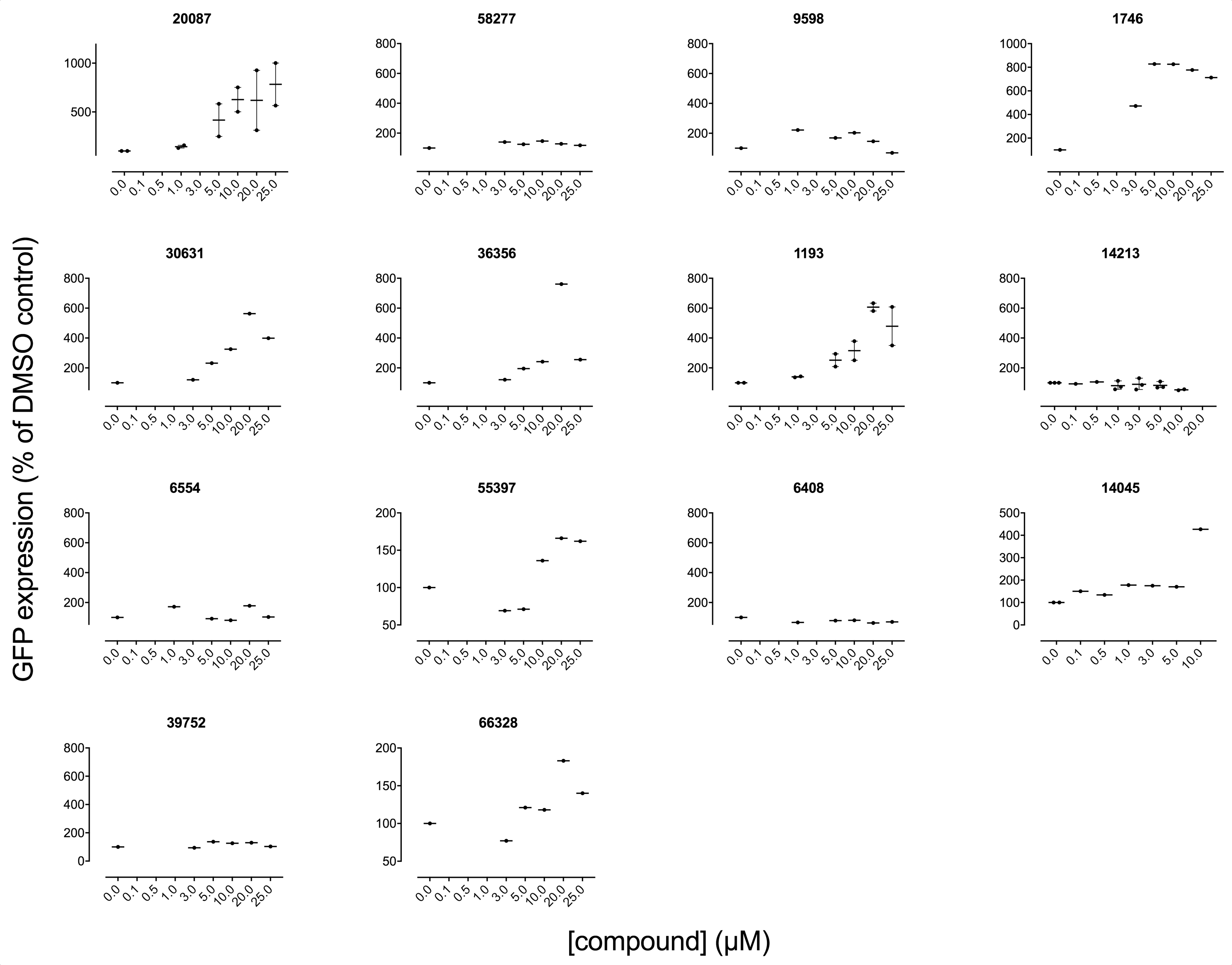

Supplement: Supplementary file 1 [file molecules-23-01691-s001.zip › molecules-327113 Supplementary Figures & Data/Supplementary Fig S2 TimtecR.tiff]

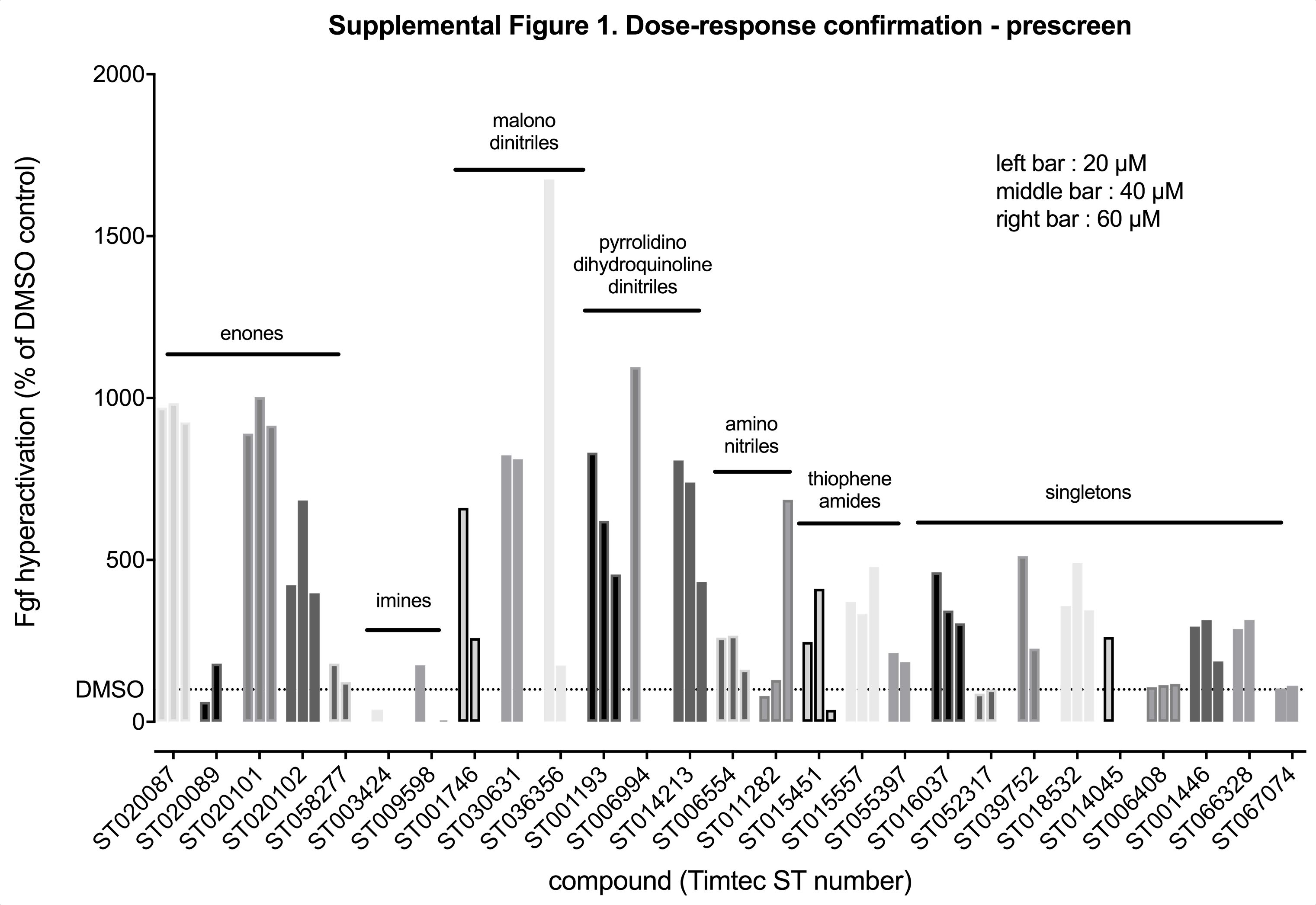

Supplement: Supplementary file 1 [file molecules-23-01691-s001.zip › molecules-327113 Supplementary Figures & Data/Supplementary Fig S1 timtecR.tiff]
